# Favorable Systemic Immune‐Inflammation Status Enhances the Neuroprotective Effects of Butylphthalide in Ischemic Stroke: A Post Hoc Analysis of BAST Trial

**DOI:** 10.1002/cns.70989

**Published:** 2026-06-22

**Authors:** Quan Zhou, Xue Tian, Xue Xia, Qin Xu, Meng Gao, Luwen Zhang, Anxin Wang

**Affiliations:** ^1^ Department of Epidemiology Beijing Neurosurgical Institute, Beijing Tiantan Hospital, Capital Medical University Beijing China; ^2^ China National Clinical Research Center for Neurological Diseases, Beijing Tiantan Hospital, Capital Medical University Beijing China; ^3^ Department of Clinical Epidemiology and Clinical Trial Capital Medical University Beijing China

**Keywords:** BAST, butylphthalide, ischemic stroke, modified Rankin scale score, network pharmacology analysis, systemic immune‐inflammation index

## Abstract

**Background:**

Among revascularized acute ischemic stroke patients, residual poor outcomes may partially arise from dysregulated poststroke inflammatory and immune responses.

**Methods:**

In this secondary analysis of the BAST trial, the systemic immune‐inflammation index (SII) was calculated for 837 patients with acute ischemic stroke who underwent revascularization at baseline and on Day 14 after treatment, and patients were categorized into four distinct SII change patterns. Network pharmacology analysis was conducted to explore potential mechanisms linking butylphthalide, inflammation‐related pathways, and ischemic stroke outcomes.

**Results:**

Compared with the persistent unfavorable group, both the unfavorable‐to‐favorable and persistent favorable groups had higher odds of a favorable modified Rankin Scale (mRS) score (odds ratio (OR) = 2.79, 95% confidence interval (CI): 1.64, 4.73, *p*_FDR_ < 0.001; OR = 2.60, 95% CI: 1.66, 4.06, *p*_FDR_ < 0.001) and an mRS score of 0–2 on Day 90. These groups also had lower risks of recurrent stroke, vascular events, and death within 90 days. Patients with persistent favorable SII who received butylphthalide showed the most favorable observed outcome profile, including the highest proportion of favorable mRS score on Day 90 (OR = 3.60, 95% CI: 1.97, 6.58, *p*_FDR_ < 0.001) and a higher proportion of mRS score 0–2 on Day 90 (OR = 4.38, 95% CI: 2.23, 8.62, *p*_FDR_ < 0.001) and the lowest risk of recurrent vascular events. Network pharmacology findings suggested that these exploratory associations may involve multi‐pathway regulation of neuroinflammation, cellular stress responses, and vascular pathology.

**Conclusions:**

Favorable or improving SII status during the acute phase of ischemic stroke was associated with better 90‐day functional outcomes and lower vascular risk. Patients with persistently favorable SII who were assigned to butylphthalide showed the most favorable observed outcome profile.

## Graphical abstract

This post hoc analysis of the BAST trial demonstrates that favorable or improving systemic immune‐inflammation index (SII) status during the acute phase of ischemic stroke was associated with better 90‐day functional outcomes and lower vascular risk. Patients with persistently favorable SII who were assigned to butylphthalide showed the most favorable observed outcome profile.

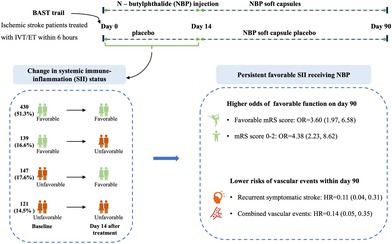

## 1 Introduction

Ischemic stroke was the third‐largest cause of disability‐adjusted life years among cardiovascular diseases globally, a significant share of which was attributable to China [1]. Ischemia‐hypoxia resulting from cerebrovascular events triggers energetic deficits, excitotoxicity, neuroinflammation, and blood–brain barrier disruption, ultimately leading to neuronal death and synaptic damage [2]. In China, approximately 12.5% (95% confidence interval (CI) 12.4%–12.5%) of stroke survivors were left disabled, as defined by a modified Rankin Scale (mRS) score greater than 1 [3]. Given this significant disease burden, intravenous thrombolysis and endovascular treatment are established reperfusion therapies delivered during the hyperacute phase of ischemic stroke [4, 5]. However, ischemia/reperfusion injury exacerbates brain damage and neurological deficits [6]. Therefore, cerebral protective agents that enhance collateral circulation compensation are also used in the acute‐phase treatment of ischemic stroke [7].

DL‐3‐*n*‐butylphthalide is a synthesized compound that was originally extracted from seeds of *Apium graveolens* (Chinese celery) [8]. Preclinical data showed that butylphthalide could play a protective role on cerebral infarction through improving microcirculation, promoting angiogenesis, and increasing cerebral blood flow in the ischemic region [9, 10]. The efficacy and safety of Butylphthalide for acute ischemic stroke patients receiving intravenous thrombolysis or endovascular treatment (BAST) trial initiated by our team revealed that among patients with acute ischemic stroke receiving intravenous thrombolysis and/or endovascular treatment, the proportions of patients with a favorable mRS score on Day 90 were 56.7% in the butylphthalide group and 44.0% in the placebo group [11]. However, despite butylphthalide treatment, 43.3% of patients with acute ischemic stroke who received intravenous thrombolysis and/or endovascular treatment still had an unfavorable mRS score at 90 days. These residual unfavorable outcomes may partly be linked to dysregulated inflammatory and immune responses [12, 13, 14]. The systemic immune‐inflammatory index (SII) is a composite marker reflecting the immune and inflammatory status of participants [15, 16]. Previous studies reported that favorable SII values on admission were associated with favorable 90‐day outcomes in acute ischemic stroke patients treated with intravenous thrombolysis and/or endovascular treatment [17, 18, 19]. Cumulated evidence supported that poststroke neuroinflammation during the acute phase is a dynamic and biphasic process [20, 21, 22, 23]. Therefore, we hypothesized that a persistently favorable inflammatory‐immune profile during the acute phase of ischemic stroke may be associated with better post‐stroke functional outcomes. We further explored whether SII change patterns and butylphthalide treatment were jointly associated with clinical outcomes.

This post hoc analysis of the BAST trial aimed to explore the associations of SII change patterns, butylphthalide treatment, and their joint categories with functional outcomes. Specifically, we assessed: (1) the association between SII change patterns and functional outcomes; (2) the association of butylphthalide treatment with functional outcomes within different SII change patterns; and (3) the joint association of SII change patterns and butylphthalide treatment with functional outcomes. Additionally, network pharmacology was used to explore potential biological pathways linking butylphthalide, inflammation‐related mechanisms, and poststroke outcomes.

## 2 Method

### 2.1 Study Design and Population

**FIGURE 1:**
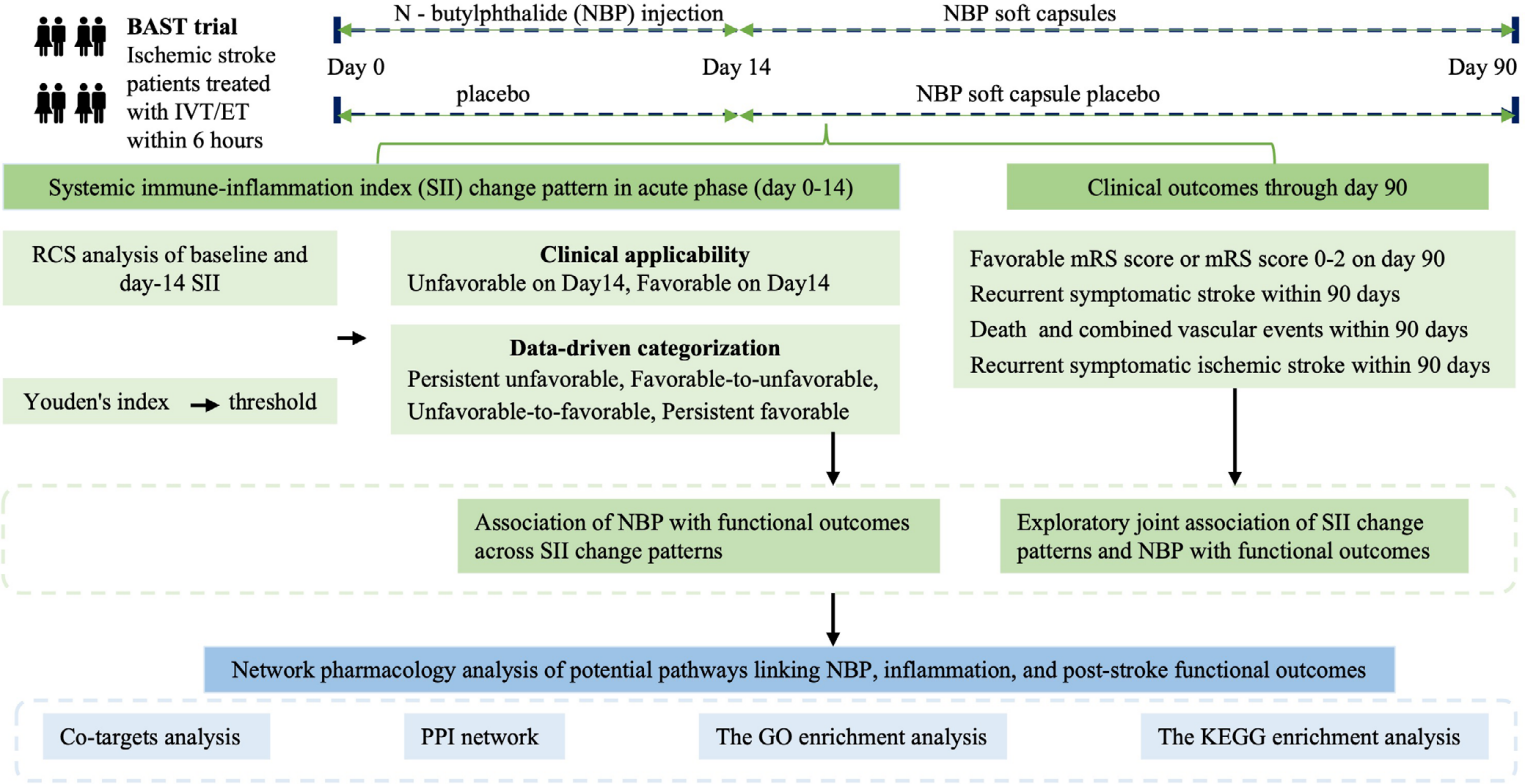
Schematic diagram of the research methodology. This figure illustrates the study design, analytical framework, and their logical relationships.

This was a post hoc analysis of the BAST trial. The study design and analytical framework are illustrated in Figure 1. Briefly, the BAST trial was a multicenter, double‐blind, placebo‐controlled, parallel randomized clinical trial at 59 centers in China, featuring a 90‐day follow‐up. It aimed to assess the efficacy and safety of butylphthalide in acute ischemic stroke patients receiving intravenous thrombolysis and/or endovascular treatment. The trial enrolled 1216 patients (≥ 18 years) with National Institutes of Health Stroke Scale (NIHSS) score of 4–25, who started the trial drug within 6 h of symptom onset after receiving intravenous thrombolysis and/or endovascular treatment. For our analysis, 379 patients were excluded for having missing or outlier SII values on baseline or Day 14 after treatment (Figure S1).

### 2.2 Randomization and Treatment

Within 6 h of symptom onset, eligible patients were randomized 1:1 to receive either butylphthalide or placebo, in addition to standard intravenous thrombolysis and/or endovascular therapy. The butylphthalide regimen consisted of intravenous butylphthalide with 100 mL sodium chloride injection twice daily for the first 14 days, followed by oral 0.2 g butylphthalide soft capsules three times daily for the subsequent 76 days. The placebo group adhered to an identical schedule, receiving matching placebo injections and capsules.

### 2.3 Measurements of Systemic Immune‐Inflammatory Index Change Patterns

The SII was derived from routine complete blood count data using the formula: SII = Platelet count (×10^9^/L) × Neutrophil count (×10^9^/L) / Lymphocyte count (×10^9^/L). The SII was measured at baseline and on Day 14 after treatment. We employed a three‐step approach to define SII change patterns of patients (Figures S2 and S3). From a clinical perspective, patients were dichotomized into two groups based on their SII status at Day 14 after treatment. Further details are provided in the Supporting Information.

### 2.4 Outcome Ascertainment

The primary efficacy outcome was the proportion of patients achieving a favorable functional outcome at 90 days post‐randomization. The definition of a favorable outcome was stratified by baseline NIHSS score: an mRS score of 0 for a baseline NIHSS of 4–7; an mRS score of 0–1 for a baseline NIHSS of 8–14; and an mRS score of 0–2 for a baseline NIHSS of 15–25 [24, 25]. Additionally, from a clinical perspective, a favorable outcome was also defined simply as an mRS score of 0 to 2 at 90 days, irrespective of the baseline NIHSS score. The secondary efficacy outcomes included recurrent symptomatic stroke within 90 days, recurrent symptomatic ischemic stroke within 90 days, combined vascular events comprising recurrent symptomatic ischemic stroke, myocardial infarction, or vascular death within 90 days. The primary safety outcome was death within 90 days in this analysis.

### 2.5 Statistical Analyses

Descriptive data were summarized as mean ± standard deviation (SD) or median (interquartile range) for continuous variables, and as counts (percentages) for categorical variables. Firstly, the associations of SII change patterns with 90‐day functional outcomes were analyzed (Table S2). Interaction between reperfusion modality and SII change patterns with 90‐day functional outcomes was also evaluated. Secondly, patients were stratified into subgroups based on their SII change patterns. Within each subgroup, the association between butylphthalide treatment, compared with placebo, and 90‐day functional outcomes was analyzed. Thirdly, to evaluate the incremental predictive value of SII change patterns, we constructed three sequential logistic regression models and compared their area under the curve (AUC) values. Interaction between baseline SII status and butylphthalide treatment with clinical outcomes (favorable mRS score on Day 90 and mRS score of 0–2 on Day 90) was evaluated to explore the effect of baseline SII on the associations between butylphthalide and outcomes. Fourth, we further explored the joint associations of SII change patterns and butylphthalide treatment with 90‐day functional outcomes including favorable mRS score and an mRS score of 0–2 on Day 90, using persistent unfavorable SII change patterns combined with placebo treatment as the reference group. We also performed the linear trend test by treating the joint category as a continuous variable. Those models adjusted age, sex, BMI, pre‐onset mRS, baseline NIHSS, and relevant medical histories (stroke, heart disease, hypertension). To correct for the multiple testing problem and decrease the risk of Type I error in the regression models, we employed the Benjamini‐Hochberg procedure to adjust raw *p*‐values based on false discovery rate [26]. To assess the robustness of the findings, sensitivity analyses were conducted for the exploratory joint associations of butylphthalide treatment and SII change patterns with favorable 90‐day mRS scores. Specifically, these analyses were performed by (1) excluding patients with acute infections or immune system diseases, and (2) additionally adjusting for reperfusion modality. All statistical analyses were performed using SAS statistical software (version 9.4) and R software (version 4.3.1). A two‐sided *p*‐value < 0.05 was considered statistically significant. Further details are provided in the Supporting Information.

### 2.6 Network Pharmacology Analysis

Network pharmacology was used to explore potential biological links among predicted butylphthalide targets, inflammation‐related targets, and ischemic‐stroke‐related targets (Table S7). Inflammation‐related targets were used as a molecular proxy for the inflammatory component represented clinically by SII; SII itself was not treated as an intervention or as a molecular target set. Subsequently, we constructed a protein–protein interaction (PPI) network using STRING database (interaction score ≥ 0.9) and analyzed network topological properties (betweenness, degree, and closeness centrality) in Cytoscape to identify key targets. Finally, we performed Gene Ontology (GO) and Kyoto Encyclopedia of Genes and Genomes (KEGG) pathway enrichment analyses to elucidate the functional roles and signaling pathways associated with these key targets. Additional details can be found in the Supporting Information.

## 3 Results

### 3.1 Characteristics of Patients

According to inclusion and exclusion criteria, a total of 837 patients from the BAST trial (female: 31.7%, median age: 66.0 years) were included in this study. Characteristics of these patients were summarized according to four SII change patterns during the acute phase of ischemic stroke (Table 1). Among 837 patients, 121 patients had persistent unfavorable SII status, and 139 patients changed from favorable to unfavorable SII status. While 147 patients changed from unfavorable to favorable and 430 patients had persistent favorable SII status. Patients with persistent unfavorable SII were more often male, older, with higher SBP, likely to be mRS score = 1 prior to onset, and likely to have a medical history of stroke, heart disease, and hypertension. Characteristics of included and excluded patients were described in Table S1. Compared with excluded patients, included patients had a lower baseline NIHSS score, whereas the other reported baseline characteristics were broadly similar.

**TABLE 1:** Characteristics of patients stratified by systemic immune‐inflammatory index change patterns during the acute phase of ischemic stroke.

Characteristics	All patients (*N* = 837)	Persistent unfavorable (*N* = 121)	Favorable−unfavorable (*N* = 139)	Unfavorable−favorable (*N* = 147)	Persistent favorable (*N* = 430)
Age, years, median (IQR)	66.0 (56.0, 72.0)	69.0 (59.0, 75.0)	69.0 (63.5, 76.0)	63.0 (54.0, 71.0)	64.0 (56.0, 71.0)
BMI, kg/m^2^, median (IQR)	24.2 (22.0, 26.4)	24.2 (22.0, 26.7)	24.0 (21.9, 26.0)	24.2 (22.3, 26.0)	24.2 (21.9, 26.7)
SBP, mmHg, median (IQR)	150.0 (137.0, 163.5)	152.5 (137.0, 166.5)	153.5 (141.2, 169.5)	142.0 (132.5, 157.8)	150.0 (137.1, 163.5)
NIHSS score, median (IQR)	8.0 (5.0, 12.0)	9.0 (5.0, 14.0)	10.0 (6.0, 13.0)	8.0 (5.5, 12.5)	7.0 (5.0, 11.0)
Female	265 (31.7%)	32 (26.4%)	35 (25.2%)	61 (41.5%)	137 (31.9%)
mRS score prior to onset = 0	780 (93.2%)	104 (86.0%)	131 (94.2%)	141 (95.9%)	404 (94.0%)
Stroke history	191 (22.8%)	36 (29.8%)	28 (20.1%)	28 (19.0%)	99 (23.0%)
Heart disease history	197 (23.5%)	31 (25.6%)	36 (25.9%)	34 (23.1%)	96 (22.3%)
Hypertension history	495 (59.1%)	79 (65.3%)	89 (64.0%)	89 (60.5%)	238 (55.3%)
Revascularization treatment					
Endovascular or bridging	251 (30.0%)	53 (43.8%)	60 (43.2%)	50 (34.0%)	88 (20.5%)
Intravenous rt‐PA treatment	586 (70.0%)	68 (56.2%)	79 (56.8%)	97 (66.0%)	342 (79.5%)
TOAST subtype					
LAA	419 (50.1%)	64 (52.9%)	73 (52.5%)	82 (55.8%)	200 (46.5%)
CE	156 (18.6%)	24 (19.8%)	38 (27.3%)	24 (16.3%)	70 (16.3%)
SAA	233 (27.8%)	25 (20.7%)	25 (18.0%)	36 (24.5%)	147 (34.2%)
SOE	13 (1.6%)	3 (2.5%)	2 (1.4%)	1 (0.7%)	7 (1.6%)
SUE	16 (1.9%)	5 (4.1%)	1 (0.7%)	4 (2.7%)	6 (1.4%)

Abbreviations: CE, cardioembolism; IQR, interquartile range; LAA, large‐artery atherosclerosis; NIHSS, National Institutes of Health Stroke Scale; SAA, small‐artery occlusion lacunar; SBP, systolic blood pressure; SOE, stroke of other determined etiology; SUE, stroke of undetermined etiology.

### 3.2 Associations of SII Change Patterns With Functional Outcomes

Compared with the persistent unfavorable group, both the unfavorable‐to‐favorable and persistent favorable groups had higher odds of achieving a favorable mRS on Day 90 (OR = 2.79, 95% CI: 1.64, 4.73, *p*_FDR_ < 0.001; OR = 2.60, 95% CI: 1.66, 4.06, *p*_FDR_ < 0.001) score and of having an mRS score of 0–2 (OR = 3.07, 95% CI: 1.66, 5.65, *p*_FDR_ < 0.001; OR = 3.14, 95% CI: 1.92, 5.12, *p*_FDR_ < 0.001) on Day 90. Similarly, these two groups had lower hazards of recurrent symptomatic stroke within 90 days (HR = 0.36, 95% CI: 0.17, 0.80, *p*_FDR_ = 0.021; HR = 0.25, 95% CI: 0.13, 0.48, *p*_FDR_ < 0.001), combined vascular events within 90 days (HR = 0.38, 95% CI: 0.18, 0.79, *p*_FDR_ = 0.020; HR = 0.27, 95% CI: 0.14, 0.50, *p*_FDR_ < 0.001) and death (HR = 0.20, 95% CI: 0.05, 0.79, *p*_FDR_ = 0.034; HR = 0.28, 95% CI: 0.11, 0.73, *p*_FDR_ = 0.020) within 90 days. The persistent favorable group had lower hazards of recurrent symptomatic ischemic stroke (HR = 0.26, 95% CI: 0.11, 0.60, *p*_FDR_ = 0.004) (Table 2). Compared with the unfavorable Day‐14 SII group, the favorable SII on Day 14 group had higher odds of favorable mRS score on Day 90 and mRS score of 0–2 on Day 90 and lower hazard of recurrent symptomatic stroke, combined vascular events, death, and recurrent symptomatic ischemic stroke within 90 days (Table S3). In subgroup analyses by revascularization modality, the unfavorable‐to‐favorable SII pattern was consistently associated with higher odds of favorable mRS score on Day 90 in both the endovascular treatment or bridging group (OR = 5.66, 95% CI: 2.07, 15.43) and the intravenous rt‐PA treatment group (OR = 2.55, 95% CI: 1.28, 5.08). Similar associations were observed for the persistent favorable SII pattern in both the endovascular treatment or bridging group (OR = 2.18, 95% CI: 1.02, 4.65) and the intravenous rt‐PA treatment group (OR = 2.97, 95% CI: 1.65, 5.38). The association between SII change patterns and favorable mRS score on Day 90 did not significantly differ by reperfusion modality (*p* for interaction = 0.321) (Table S5).

**TABLE 2:** Associations of systemic immune‐inflammation change patterns with 90‐day functional outcomes in patients with ischemic stroke.

Functional outcomes	Systemic immune inflammation change pattern	Events/total (proportion)	Adjusted effect estimate (95% CI)	*p*_FDR_ value
Favorable mRS score on Day 90	Persistent unfavorable	45/121 (37.2%)	OR = 1 [Reference]	
	Favorable−unfavorable	49/139 (35.3%)	0.9 (0.53, 1.53)	0.698
	Unfavorable−favorable	94/147 (63.9%)	**2.79 (1.64, 4.73)**	**< 0.001**
	Persistent favorable	255/430 (59.3%)	**2.60 (1.66, 4.06)**	**< 0.001**
mRS score of 0–2 on Day 90	Persistent unfavorable	72/121 (59.5%)	OR = 1 [Reference]	
	Favorable−unfavorable	75/139 (54.0%)	0.76 (0.45, 1.3)	0.434
	Unfavorable−favorable	123/147 (83.7%)	**3.07 (1.66, 5.65)**	**< 0.001**
	Persistent favorable	368/430 (85.6%)	**3.14 (1.92, 5.12)**	**< 0.001**
Recurrent symptomatic stroke within 90 days	Persistent unfavorable	19/121 (15.7%)	HR = 1 [Reference]	
	Favorable−unfavorable	19/139 (13.7%)	0.84 (0.44, 1.59)	0.623
	Unfavorable−favorable	10/147 (6.8%)	**0.36 (0.17, 0.80)**	**0.021**
	Persistent favorable	20/430 (4.7%)	**0.25 (0.13, 0.48)**	**< 0.001**
Combined vascular events within 90 days	Persistent unfavorable	21/121 (17.4%)	HR = 1 [Reference]	
	Favorable−unfavorable	21/139 (15.1%)	0.83 (0.45, 1.54)	0.623
	Unfavorable−favorable	11/147 (7.5%)	**0.38 (0.18, 0.79)**	**0.020**
	Persistent favorable	22/430 (5.1%)	**0.27 (0.14, 0.50)**	**< 0.001**
Death within 90 days	Persistent unfavorable	9/121 (7.4%)	HR = 1 [Reference]	
	Favorable−unfavorable	10/139 (7.2%)	0.79 (0.35, 1.80)	0.623
	Unfavorable−favorable	2/147 (1.4%)	**0.20 (0.05, 0.79)**	**0.034**
	Persistent favorable	7/430 (1.6%)	**0.28 (0.11, 0.73)**	**0.020**
Recurrent symptomatic ischemic stroke within 90 days	Persistent unfavorable	11/121 (9.1%)	HR = 1 [Reference]	
	Favorable−unfavorable	7/139 (5.0%)	0.52 (0.20, 1.36)	0.275
	Unfavorable−favorable	10/147 (6.8%)	0.70 (0.29, 1.69)	0.554
	Persistent favorable	12/430 (2.8%)	**0.26 (0.11, 0.60)**	**0.004**

*Note:* For all models, the “persistent unfavorable” pattern served as the reference category, with adjustments made for baseline covariates including treatment (butylphthalide vs. placebo), age, sex, body mass index, pre‐onset modified Rankin Scale score, National Institutes of Health Stroke Scale score, and relevant medical histories (stroke, heart disease, hypertension). Boldface indicates statistically significant associations (*p*_FDR_ < 0.05).

Abbreviations: HR: hazard ratio; OR: odds ratio; *p*_FDR_ value: false discovery rate‐adjusted *p*‐value.

### 3.3 Associations of Butylphthalide Treatment With Functional Outcomes Across Different SII Change Patterns

The associations of butylphthalide treatment with 90‐day functional outcomes across different SII change patterns and by SII status at Day 14 post‐treatment are presented in Tables S4 and S6. Butylphthalide treatment was associated with higher odds of achieving a favorable mRS score on Day 90 among patients with persistent favorable SII status (OR = 1.83, 95% CI: 1.21, 2.77; *p*_FDR_ = 0.044) and among those with favorable SII status at Day 14 posttreatment (OR = 1.83, 95% CI: 1.28, 2.63; *p*_FDR_ = 0.012). No significant interaction was observed between butylphthalide treatment and SII change patterns for either functional outcome (both *p* for interaction > 0.05). Similarly, no significant interaction was observed between butylphthalide treatment and Day‐14 SII status for either functional outcome (both *p* for interaction > 0.05). ROC curve analyses showed that incorporating SII change patterns, either alone or as joint categories with butylphthalide treatment, improved the predictive performance for 90‐day functional outcomes compared with the baseline model, which included butylphthalide treatment, age, sex, body mass index, pre‐onset mRS, baseline NIHSS score, and relevant medical histories, including stroke, heart disease, and hypertension (*p* < 0.01). Specifically, the AUC increased from 0.661 to 0.706 for favorable mRS score on Day 90 and from 0.729 to 0.771 for mRS score of 0–2 on Day 90 after adding SII change patterns, indicating improved model discrimination in this dataset (Figure S4). As shown in Figure S10, no significant interaction was observed between baseline SII status and butylphthalide treatment for either favorable mRS score or mRS score of 0–2 on Day 90 (both *p* for interaction > 0.05).

### 3.4 Exploratory Joint Associations of Butylphthalide Treatment and SII Change Patterns With Functional Outcomes

**FIGURE 2:**
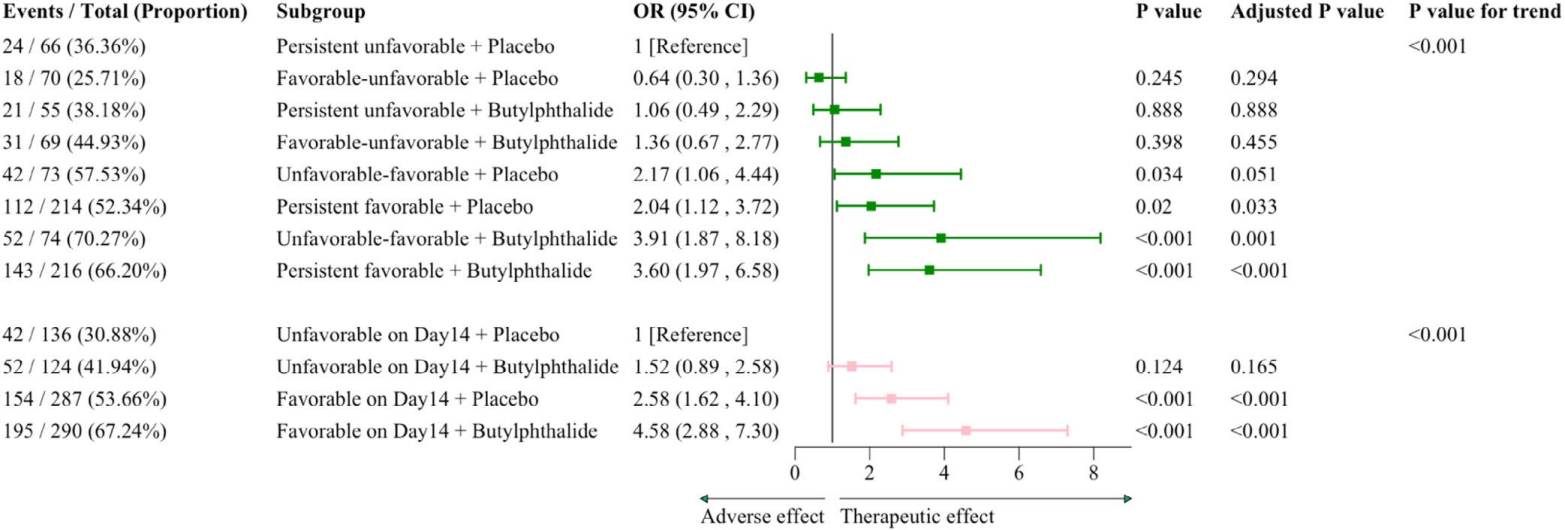
Exploratory joint associations of butylphthalide treatment and systemic immune‐inflammation index change patterns with favorable mRS score on Day 90 in patients with ischemic stroke. In the analysis examining the exploratory joint association of butylphthalide treatment and SII change patterns with favorable mRS score on Day 90, the “Persistent unfavorable + Placebo” pattern served as the reference category. In the analysis examining joint associations of butylphthalide and SII status with Day 14 post‐treatment with favorable mRS score on Day 90, the “Unfavorable + Placebo” pattern served as the reference category. Both analyses were adjusted for baseline covariates, including age, sex, body mass index, pre‐onset modified Rankin Scale score, National Institutes of Health Stroke Scale score, and relevant medical histories (stroke, heart disease, hypertension). OR, odds ratio; *p*_FDR_ value, false discovery rate‐adjusted *p*‐value.

The exploratory joint associations of butylphthalide treatment and SII change patterns with 90‐day functional outcomes in patients with ischemic stroke are reported. We observed a generally joint association of butylphthalide treatments and changes in SII patterns (from persistent unfavorable to persistent favorable) with a higher rate of favorable mRS score on Day 90 (Figure 2), mRS score of 0–2 on Day 90 (Figure S5), and lower risk of recurrent symptomatic stroke within 90 days (Figure S6) along with combined vascular events within 90 days (Figure S7) (all *p* for linear trend < 0.001). Compared with those with persistent unfavorable SII patterns and placebo treatment, participants with persistent favorable SII patterns and butylphthalide had a higher proportion of favorable mRS score on Day 90 (OR = 3.60, 95% CI: 1.97, 6.58; *p*_FDR_ < 0.001) and higher proportion of mRS score 0–2 on Day 90 (OR = 4.38, 95% CI: 2.23, 8.62; *p*_FDR_ < 0.001) (Figures 2 and S5). Compared with those with unfavorable SII status at Day 14 post‐treatment and placebo treatment, participants with favorable SII status at Day 14 post‐treatment and butylphthalide had a higher proportion of favorable mRS score on Day 90 (OR = 4.58, 95% CI: 2.88, 7.30; *p*_FDR_ < 0.001) and higher proportion of mRS score 0–2 on Day 90 (OR = 5.67, 95% CI: 3.39, 9.51; *p*_FDR_ < 0.001) (Figures 2 and S5). Compared with those with persistent unfavorable SII patterns and placebo treatment, participants with persistent favorable SII patterns and butylphthalide had lower risk of recurrent symptomatic stroke (HR = 0.11, 95% CI: 0.04, 0.31; *p*_FDR_ < 0.001) along with combined vascular events within 90 days (HR = 0.14, 95% CI: 0.05, 0.35; *p*_FDR_ < 0.001) (Figures S6 and S7). Compared with those with unfavorable SII status at Day 14 posttreatment and placebo treatment, participants with favorable SII status at Day 14 posttreatment and butylphthalide had lower risk of recurrent symptomatic stroke along with combined vascular events within 90 days (Figures S6 and S7). Sensitivity analyses further indicated that the exploratory joint associations of butylphthalide treatment and SII change patterns with favorable 90‐day functional outcomes (mRS scores) remained stable after further excluding patients with acute infections or immune system diseases (Figure S8) and after additional adjustment for reperfusion modality (Figure S9).

### 3.5 Network Pharmacology Analysis of Potential Pathways Linking Butylphthalide, Inflammation, and Post‐Stroke Functional Outcomes

**FIGURE 3:**
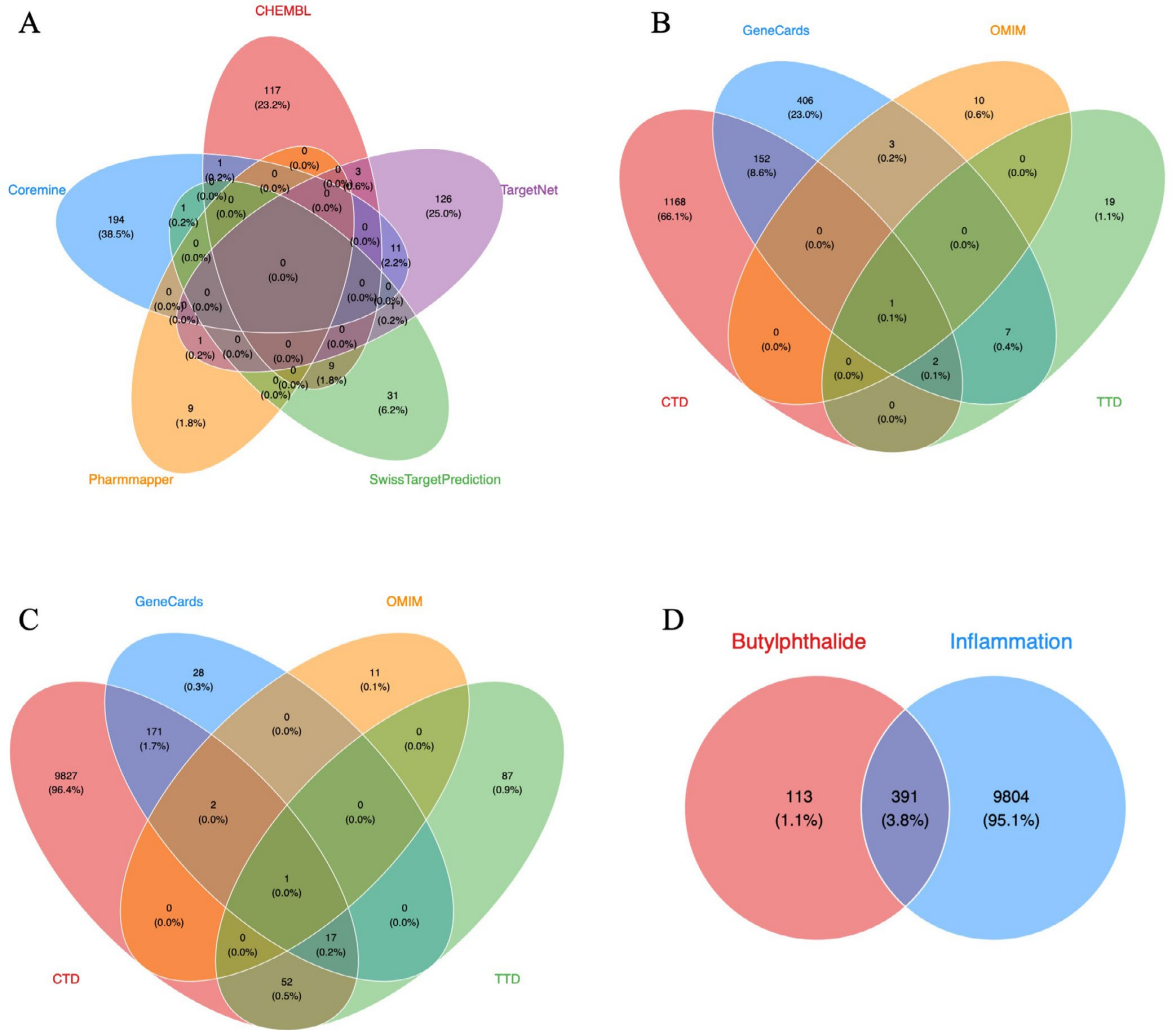
Venn diagram for gene targets. (A) Venn diagram for gene targets of butylphthalide; (B) Venn diagram for gene targets of ischemic stroke; (C) Venn diagram for gene targets of inflammation; (D) Venn diagram for co‐gene targets of butylphthalide and inflammation.

**FIGURE 4:**
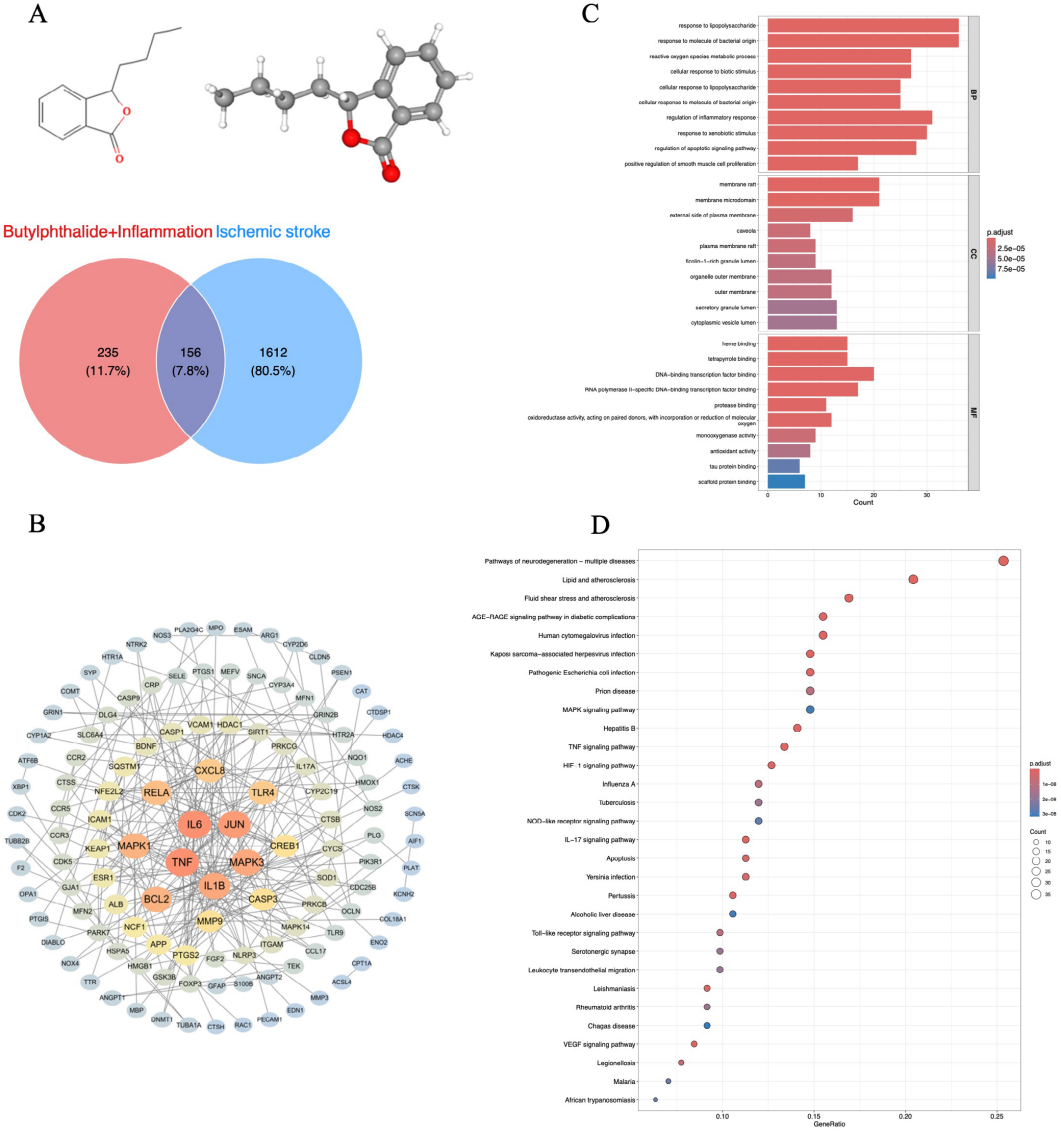
Enrichment analysis and PPI network of targets shared by butylphthalide, inflammation, and ischemic stroke. (A) The structure of butylphthalide and a Venn diagram of targets shared by butylphthalide, inflammation, and ischemic stroke. (B) PPI network of co‐targets. (C) GO enrichment analysis of co‐targets. (D) KEGG enrichment analysis of co‐targets.

After the screening, merging, comparing, and de‐redundancy of the data collected from databases, 504 human gene targets of butylphthalide, 10,195 human gene targets of inflammation, 1768 human gene targets of ischemic stroke and 156 butylphthalide—inflammation—ischemic stroke co‐targets were identified (Figures 3 and 4A). The two‐ and three‐dimensional structures of butylphthalide were obtained from PubChem (Figure 4A). We analyzed the PPI network of 156 targets shared by butylphthalide, inflammation, and ischemic stroke using the STRING database and formed a PPI network with 156 nodes (targets), 261 edges (interactions between target proteins), and node degree values ranging from 0 to 17. The nodes' degree values were displayed in color shades and sizes to visualize the PPI network (Figure 4B). We also performed enrichment analysis using the DAVID database. The results showed 2253 GO‐BP entries, 64 GO‐CC entries, 121 GO‐MF entries, and 170 KEGG pathways were enriched among 156 drug‐disease co‐targets. The co‐targets were mainly enriched in the biological processes of inflammatory response, response to oxidative stress, and cellular response to chemical stimuli; the cellular components of membrane rafts and membrane microdomains; and the molecular functions of protein binding and transcription factor binding (Figure 4C). KEGG pathway enrichment analysis revealed that the co‐targets were predominantly enriched in pathways of neurodegeneration, lipid and atherosclerosis, and fluid shear stress. Key inflammatory and stress‐related pathways, including the tumor necrosis factor (TNF) signaling pathway and hypoxia‐inducible factor 1 (HIF‐1) signaling pathway, were also significantly represented. These findings suggest that the exploratory associations among butylphthalide treatment, inflammatory status, and ischemic stroke outcomes may involve multi‐pathway regulation of neuroinflammation, cellular stress responses, and vascular pathology (Figure 4D).

## 4 Discussion

In this post hoc analysis of the BAST trial, the impact of dynamic SII patterns, butylphthalide treatment, and their joint effects on 90‐day functional outcomes in patients with acute ischemic stroke receiving intravenous thrombolysis and/or endovascular treatment were investigated. First, compared to patients with persistent unfavorable SII, those whose SII improved from unfavorable to favorable or remained persistently favorable had significantly better functional outcomes at 90 days, along with a reduced risk of stroke recurrence, composite vascular events, and death within 90 days. Second, patients with persistently favorable SII who were assigned to butylphthalide had the most favorable observed outcome profile in exploratory joint‐category analyses; however, formal interaction tests did not show statistically significant treatment‐effect modification for the functional outcomes. However, incorporating SII into the predictive model significantly enhanced the accuracy for predicting 90‐day functional outcomes. Network pharmacology analysis further suggested that these exploratory associations may involve multiple pathways, including neuroinflammation, oxidative stress, and vascular pathology.

Our findings suggest that patients with an improving (from unfavorable to favorable) or persistently favorable SII pattern during the acute phase had significantly better 90‐day functional outcomes in patients with acute ischemic stroke receiving intravenous thrombolysis and/or endovascular treatment. This finding aligns with numerous previous studies indicating that a lower SII, reflecting a less severe systemic inflammatory state, is associated with improved functional recovery in acute ischemic stroke patients receiving intravenous thrombolysis and/or endovascular treatment [17, 18, 27]. Our study extended the investigation by calculating SII not only at admission but also at Day 14 post‐treatment and defining the dynamic SII change patterns during the acute phase (0–14 days after symptom onset) by using ROC analysis. Beyond its association with 90‐day functional outcomes, a favorable SII pattern was also associated with a significantly reduced risk of stroke recurrence, composite vascular events, and all‐cause mortality within 90 days.

A key finding of our study is the joint association of butylphthalide treatment and SII change pattern with functional outcomes. Specifically, butylphthalide treatment was significantly associated with better functional outcomes at 90 days, along with reduced risk of stroke recurrence, composite vascular events, and death at 90 days only in the subgroup of patients with persistently favorable SII pattern or from unfavorable to favorable SII pattern. Furthermore, no significant interaction was found between SII change patterns and butylphthalide treatment. Our finding resonates with the hypothesis that the neuroprotective effectiveness of butylphthalide may be influenced by the post‐stroke inflammatory milieu. Moreover, ROC curve analysis demonstrated that incorporating SII, either alone or in combination with butylphthalide, significantly enhanced the predictive accuracy for 90‐day favorable functional outcomes which was consistent with the results of previous studies [18].

We observed an exploratory joint association of butylphthalide treatment and favorable SII status with post‐stroke functional outcomes. Compared to patients with persistent unfavorable SII and receiving placebo, those with persistent favorable SII and receiving butylphthalide had the highest proportion of favorable mRS scores and mRS scores of 0–2 at 90 days and lower risk of recurrent symptomatic stroke along with combined vascular events within 90 days. The network pharmacology analysis identified 156 overlapping targets related to butylphthalide, inflammation, and ischemic stroke. These co‐targets were significantly enriched in pathways related to neuroinflammation (e.g., TNF signaling pathway), response to oxidative stress (e.g., HIF‐1 signaling pathway), and atherosclerosis. While clinical evidence remains scarce, our findings are supported by relevant preclinical studies in animal models. Previous studies have confirmed that butylphthalide possesses anti‐inflammatory, antioxidant, and anti‐apoptotic properties. An analysis involving 13 adult male cynomolgus monkeys found that butylphthalide improves working memory by alleviating remote secondary neurodegeneration and neuroinflammation in the ipsilateral dorsal lateral prefrontal cortex and thalamus after middle cerebral artery occlusion in cynomolgus monkeys [28]. An animal experiment conducted on 60 male Sprague–Dawley rats revealed that butylphthalide treatment alleviated brain injury induced by cerebral ischemia–reperfusion through modulating the hepatocyte‐growth factor‐regulated TLR4/NF‐κB inflammatory pathway [29]. Our integrated findings, combining exploratory clinical observations and network pharmacology predictions, suggest a potential association between SII dynamics and functional outcomes in patients treated with butylphthalide. Patients receiving butylphthalide with a favorable SII profile showed the most favorable observed outcome pattern; however, formal interaction analyses did not confirm treatment‐effect heterogeneity. These findings should therefore be considered hypothesis‐generating. Future prospective studies are needed to validate SII dynamics as a potential biomarker associated with butylphthalide‐related outcomes.

Our study has several limitations. First, given the post hoc nature of this analysis and its limited statistical power, our findings should be interpreted as hypothesis‐generating, warranting validation through subsequent prospective studies. Second, the immune‐inflammatory status was assessed using the SII rather than high‐sensitivity C‐reactive protein or immunoglobulins. However, the SII is more readily accessible in clinical practice and is supported by extensive evidence linking it to functional outcomes in ischemic stroke patients. Third, the results of the network pharmacology analysis are based on theoretical predictions derived from existing databases; the specific molecular mechanisms require further validation through in vitro and in vivo biological experiments. Fourth, this study exclusively enrolled a Chinese population, which may limit the generalizability of the findings to other ethnic groups. Fifth, although we employed the Benjamini–Hochberg procedure to adjust raw *p*‐values based on the false discovery rate to correct for multiple testing, the potential for Type I error remains a consideration. Sixth, SII was measured only at hospitalization and 14 days post‐treatment, which restricts the characterization of subacute SII dynamics after ischemic stroke onset. In addition, SII change patterns were defined using Day‐14 SII, a post‐randomization variable; therefore, analyses conditioned on SII changes should be interpreted as exploratory associations rather than evidence for causal treatment stratification.

## 5 Conclusion

This post hoc analysis of the BAST trial demonstrates that favorable or improving SII status during the acute phase of ischemic stroke was associated with better 90‐day functional outcomes and lower vascular risk. Patients with persistently favorable SII who were assigned to butylphthalide showed the most favorable observed outcome profile, while formal interaction analyses did not confirm treatment‐effect heterogeneity. Network pharmacology findings suggested that these exploratory associations may involve inflammation‐related and vascular pathways. These findings are hypothesis‐generating and support further prospective studies to evaluate the clinical utility of SII monitoring in patients treated with butylphthalide.

## Author Contributions

Conceptualization: Quan Zhou and Anxin Wang. Study design: Quan Zhou and Anxin Wang. Methodology: Quan Zhou, Xue Tian, Qin Xu, Xue Xia, Meng Gao, Luwen Zhang, and Anxin Wang. Data collection: Quan Zhou, Qin Xu, Xue Xia, and Anxin Wang. Investigation: Quan Zhou, Qin Xu, Xue Xia, and Anxin Wang. Statistical analysis: Quan Zhou. Writing‐original draft: Quan Zhou. Writing‐review and editing: Quan Zhou, Xue Tian, Xue Xia, Qin Xu, Meng Gao, Luwen Zhang, and Anxin Wang. Funding acquisition: Quan Zhou, Anxin Wang. All authors approved the final manuscript.

## Funding

This work was supported by the Outstanding Young Investigator Program of Capital Medical University (grant number A2404), National Key Technology Research and Development Program of the Ministry of Science and Technology of the People's Republic of China (grant number 2016YFC1301501), and Beijing Neurosurgical Institute Youth Innovation Fund (grant number 5).

## Ethics Statement

The trial protocol was approved by the Ethics Committee of Beijing Tiantan Hospital (institutional review board approval number: KY 2018‐003‐08) and all participating centers. The trial was registered at ClinicalTrials.gov (Registration URL: http://www.clinicaltrials.gov) under the unique identifier NCT03539445.

## Consent

All participants or their representatives provided written informed consent prior to enrollment.

## Conflicts of Interest

The authors declare no conflicts of interest.

## Supporting information


**Figure S1:** Selection process of the study population.
**Figure S2:** Restricted cubic splines of the systemic immune‐inflammation index for favorable mRS and mRS score of 0–2 on Day 90.
**Figure S3:** Four SII change patterns during acute phase of ischemic stroke stratified by treatment.
**Table S1:** Baseline characteristics of included and excluded patients.
**Table S2:** Proportional hazards assumption test results for the Cox model of systemic immune inflammation related indicator with outcomes.
**Table S3:** Associations of Day‐14 systemic immune‐inflammation status with 90‐day functional outcomes in patients with ischemic stroke.
**Table S4:** Associations of butylphthalide treatment with 90‐day functional outcomes across different systemic immune‐inflammation change patterns.
**Table S5:** Associations of systemic immune inflammation change patterns with a favorable mRS score on Day 90 stratified by reperfusion modality.
**Table S6:** Associations of butylphthalide treatment with 90‐day functional outcomes by Day‐14 systemic immune‐inflammation status.
**Figure S4:** ROC curve analysis of regression models for evaluating the predictive performance enhancement by the systemic immune‐inflammation index change patterns.
**Figure S5:** Joint associations of butylphthalide and systemic immune‐inflammation index change pattern with a mRS score of 0–2 on Day 90.
**Figure S6:** Joint associations of butylphthalide and systemic immune‐inflammation index change pattern with recurrent symptomatic stroke within 90 days.
**Figure S7:** Joint associations of butylphthalide and systemic immune‐inflammation index change pattern with combined vascular events within 90 days.
**Figure S8:** Sensitivity analysis of the joint associations of butylphthalide and systemic immune‐inflammation index change patterns with a favorable 90‐day mRS scores in ischemic stroke patients additionally excluding individuals with acute infection or immune system diseases.
**Figure S9:** Sensitivity analysis of the joint associations of butylphthalide and SII change patterns with a favorable 90‐day mRS scores in ischemic stroke patients additionally adjusting reperfusion modality.
**Figure S10:** Interaction between baseline SII value and butylphthalide treatment on 90‐day functional outcomes.
**Table S7:** Detailed information on identification of gene targets.
**Supporting Information:** method.

**Data S1:** STROBE Statement—checklist of items that should be included in reports of observational studies.

## Data Availability

The data that support the findings of this study are available on request from the corresponding author. The data are not publicly available due to privacy or ethical restrictions.
