# Supplementary material for: Favorable Systemic Immune‐Inflammation Status Enhances the Neuroprotective Effects of Butylphthalide in Ischemic Stroke: A Post Hoc Analysis of BAST Trial
Source: CNS Neurosci Ther. 2026 Jun 22;32(6):e70989. doi: 10.1002/cns.70989 (PMC13285582; doi:10.1002/cns.70989)
Supplement: Supplementary file 1 — Figure S1: Selection process of the study population. Figure S2: Restricted cubic splines of the systemic immune‐inflammation index for favorable mRS and mRS score of 0–2 on Day 90. Figure S3: Four SII change patterns during acute phase of ischemic stroke stratified by treatment. Table S1: Baseline characteristics of included and excluded patients. Table S2: Proportional hazards assumption test results for the Cox model of systemic immune inflammation related indicator with outcomes. Table S3: Associations of Day‐14 systemic immune‐inflammation status with 90‐day functional outcomes in patients with ischemic stroke. Table S4: Associations of butylphthalide treatment with 90‐day functional outcomes across different systemic immune‐inflammation change patterns. Table S5: Associations of systemic immune inflammation change patterns with a favorable mRS score on Day 90 stratified by reperfusion modality. Table S6: Associations of butylphthalide treatment with 90‐day functional outcomes by Day‐14 systemic immune‐inflammation status. Figure S4: ROC curve analysis of regression models for evaluating the predictive performance enhancement by the systemic immune‐inflammation index change patterns. Figure S5: Joint associations of butylphthalide and systemic immune‐inflammation index change pattern with a mRS score of 0–2 on Day 90. Figure S6: Joint associations of butylphthalide and systemic immune‐inflammation index change pattern with recurrent symptomatic stroke within 90 days. Figure S7: Joint associations of butylphthalide and systemic immune‐inflammation index change pattern with combined vascular events within 90 days. Figure S8: Sensitivity analysis of the joint associations of butylphthalide and systemic immune‐inflammation index change patterns with a favorable 90‐day mRS scores in ischemic stroke patients additionally excluding individuals with acute infection or immune system diseases. Figure S9: Sensitivity analysis of the joint associations of butylphthalide an [file CNS-32-e70989-s001.docx]

Table of Contents

[Supplementary method 3](#_Toc231750312)

[Figure S1. Selection process of the study population 7](#_Toc231750313)

[Figure S2. Restricted cubic splines of the systemic immune-inflammation index for favorable mRS and mRS score of 0-2 on day 90 8](#_Toc231750314)

[Figure S3. Four SII change patterns during acute phase of ischemic stroke stratified by treatment 9](#_Toc231750315)

[Table S1. Baseline characteristics of included and excluded patients 10](#_Toc231750316)

[Table S2. Proportional hazards assumption test results for the Cox model of systemic immune inflammation related indicator with outcomes 11](#_Toc231750317)

[Table S3. Associations of day-14 systemic immune-inflammation status with 90-day functional outcomes in patients with ischemic stroke 12](#_Toc231750318)

[Table S4. Associations of butylphthalide treatment with 90-day functional outcomes across different systemic immune-inflammation change patterns 13](#_Toc231750319)

[Table S5. Associations of systemic immune inflammation change patterns with a favorable mRS score on day 90 stratified by reperfusion modality 14](#_Toc231750320)

[Table S6. Associations of butylphthalide treatment with 90-day functional outcomes by day-14 systemic immune-inflammation status 15](#_Toc231750321)

[Figure S4. ROC curve analysis of regression models for evaluating the predictive performance enhancement by the systemic immune-inflammation index change patterns 16](#_Toc231750322)

[Figure S5. Joint associations of butylphthalide and systemic immune-inflammation index change pattern with a mRS score of 0-2 on day 90 17](#_Toc231750323)

[Figure S6. Joint associations of butylphthalide and systemic immune-inflammation index change pattern with recurrent symptomatic stroke within 90 days 18](#_Toc231750324)

[Figure S7. Joint associations of butylphthalide and systemic immune-inflammation index change pattern with combined vascular events within 90 days 19](#_Toc231750325)

[Figure S8. Sensitivity analysis of the joint associations of butylphthalide and systemic immune-inflammation index change patterns with a favorable 90-day mRS scores in ischemic stroke patients additionally excluding individuals with acute infection or immune system diseases 20](#_Toc231750326)

[Figure S9. Sensitivity analysis of the joint associations of butylphthalide and SII change patterns with a favorable 90-day mRS scores in ischemic stroke patients additionally adjusting reperfusion modality 21](#_Toc231750327)

[Figure S10. Interaction between baseline SII value and butylphthalide treatment on 90-day functional outcomes 22](#_Toc231750328)

[Table S7. Detailed information on identification of gene targets 23](#_Toc231750329)

**Supplementary method**

Measurements of systemic immuno-inflammatory index change patterns

The SII is a composite marker reflecting the immune and inflammatory status of participants and was reported to be associated with functional outcome of acute ischemic stroke patients treated with intravenous thrombolysis ^1, 2^. It was derived from routine complete blood count data using the formula: SII = Platelet count (×10⁹/L) × Neutrophil count (×10⁹/L) / Lymphocyte count (×10⁹/L). SII was measured at baseline and on day 14 after treatment. We employed a three-step approach to estimate SII change patterns of patients. Firstly, restricted cubic spline (RCS) analyses were conducted to delineate the non-linear associations of SII at baseline and on day 14 following treatment with favorable mRS score and an mRS score of 0-2 on day 90 (**Figure S2**). The results indicated that the association between SII and the primary outcomes was significant only at day 14 post-treatment, with no significant association detected at baseline. Secondly, receiver operating characteristic (ROC) curves were constructed to assess the ability of the day-14 SII to predict the favorable mRS score on day 90. We identified a post hoc, data-derived cut-off of SII (895.78 ×10⁹/L) based on the Youden's index in the same dataset. Thirdly, patients with an SII value at or above this post hoc cut-off were classified as having an unfavorable systemic immune-inflammatory status, while all others were classified as favorable. The same cut-off was applied to categorize baseline SII. Based on the SII status at baseline and day 14 after treatment, patients were categorized into one of four distinct SII change patterns during the acute stroke phase: 1) persistent unfavorable, 2) favorable-to-unfavorable, 3) unfavorable-to-favorable, and 4) persistent favorable (**Figure S3**). From a clinical perspective, patients were dichotomized into two groups based on their SII status at day 14 after treatment: those with a favorable SII status and those with an unfavorable SII status at day 14 after treatment. This post hoc cut-off has not been externally validated and may be subject to optimism and overfitting.

Statistical analyses

Firstly, the associations of SII change patterns with 90-day functional outcomes were analyzed. Specifically, multivariable logistic regression models were used to assess relationships of SII change patterns with favorable mRS score and mRS score of 0-2 on day 90. For time-to-event outcomes, including recurrent symptomatic stroke, combined vascular events, death, and recurrent symptomatic ischemic stroke within 90 days, the Cox proportional hazards regressions were fitted. Prior to conducting Cox regression, the proportional hazards assumption was tested globally using Schoenfeld residuals (**Table S2**). A significant violation (global test p < 0.05) prompted the use of a time-dependent Cox model ^3^. For all models, the "persistent unfavorable" pattern served as the reference category, with adjustments made for baseline covariates including treatment (butylphthalide vs placebo), age, sex, body mass index (BMI), pre-onset mRS, baseline NIHSS, and relevant medical histories (stroke, heart disease, hypertension) ^4-6^. The association of day-14 SII status with the functional outcomes was evaluated using the same statistical modeling strategy as described above.

Secondly, patients were stratified into subgroups based on their SII change patterns. Within each subgroup, the association between butylphthalide treatment (compared with placebo) and 90-day functional outcomes was analyzed. Multivariable logistic regression was employed to assess the association between butylphthalide on favorable mRS score and an mRS score of 0-2 on 90-day. Cox proportional hazards regression models were used to explore the association between butylphthalide on the time-to-event outcomes including recurrent symptomatic stroke, combined vascular events, death, and recurrent symptomatic ischemic stroke within 90 days, with the assumption of proportional hazards being verified. Changes in the NIHSS score from baseline to 90 days were analyzed using the Wilcoxon rank-sum test to compare distributions between treatment groups (butylphthalide versus placebo), with the Hodges-Lehmann estimator used to calculate the median difference. Those models adjusted age, sex, BMI, pre-onset mRS, baseline NIHSS, and relevant medical histories (stroke, heart disease, hypertension). Interaction between butylphthalide and SII change pattern was assessed by conducting likelihood ratio tests. The association between butylphthalide treatment (compared with placebo) was assessed separately in patients with favorable and unfavorable SII status at day 14 after treatment, using the same statistical modeling strategy as described above.

Thirdly, to evaluate the incremental predictive value of SII change patterns, we constructed three sequential logistic regression models and compared their Area Under the Curve (AUC) values. The DeLong test was employed to assess the statistical significance of the differences in predictive accuracy between the baseline model and those incorporating SII or the butylphthalide-SII combination. Fourth, we further explored the joint associations of SII change patterns and butylphthalide treatment with 90-day functional outcomes including favorable mRS score and an mRS score of 0-2 on 90-day, using persistent unfavorable SII change patterns combined with placebo treatment as the reference group. We also performed the linear trend test by treating the joint category as a continuous variable. Those models adjusted age, sex, BMI, pre-onset mRS, baseline NIHSS, and relevant medical histories (stroke, heart disease, hypertension).

Network pharmacology analysis

The PubChem database was searched to obtain two and three-dimensional structures and SMILES of butylphthalide (PubChem CID: 61,361; SMILES: CCCCC1C2=CC=CC=C2C(=O)O1; Synonym: 3-n-butylphthalide). The Coremine database, ChEMBL, TargetNet, SwissTargetPrediction and PharmMapper databases were used to predict the butylphthalide targets. The UniProt database (https://www.uniprot.org/) was used to obtain gene names for comparison. The predicted targets of butylphthalide from five datasets were merged and duplicate targets were removed. We searched for “ischemic stroke” and “inflammation” targets at Comparative Toxicogenomics Database (CTD), GeneCards, Therapeutic Target Database (TTD) and OMIM database. We curated the retrieved target genes by merging duplicates and normalizing gene names using the UniProt database (https://www.uniprot.org/). Given that butylphthalide has been implicated in inflammation-related pathways and SII is a biomarker of systemic inflammation, the overlapping targets of “butylphthalide” and “inflammation” were used to explore potential biological pathways linking butylphthalide treatment and inflammatory status ^7, 8^. Further details are provided in **Table S7**.

The Venn diagram was constructed to identify the co-targets of the butylphthalide drug target gene, inflammation set and the ischemic stroke disease target gene set. The intersecting target genes were imported into the STRING database (version 12.0) and the minimum required interaction score was set 0.900 to construct a protein–protein interaction (PPI) network. Following the analysis of network topological properties of the PPI network in Cytoscape (version 3.9), key targets for the combined butylphthalide and inflammatory status of ischemic stroke were identified based on betweenness, degree, and closeness centrality. Subsequent Gene Ontology (GO) and Kyoto Encyclopedia of Genes and Genomes (KEGG) pathway enrichment analyses for these key targets were conducted and visualized using R software to elucidate their functional roles and associated signaling pathways.


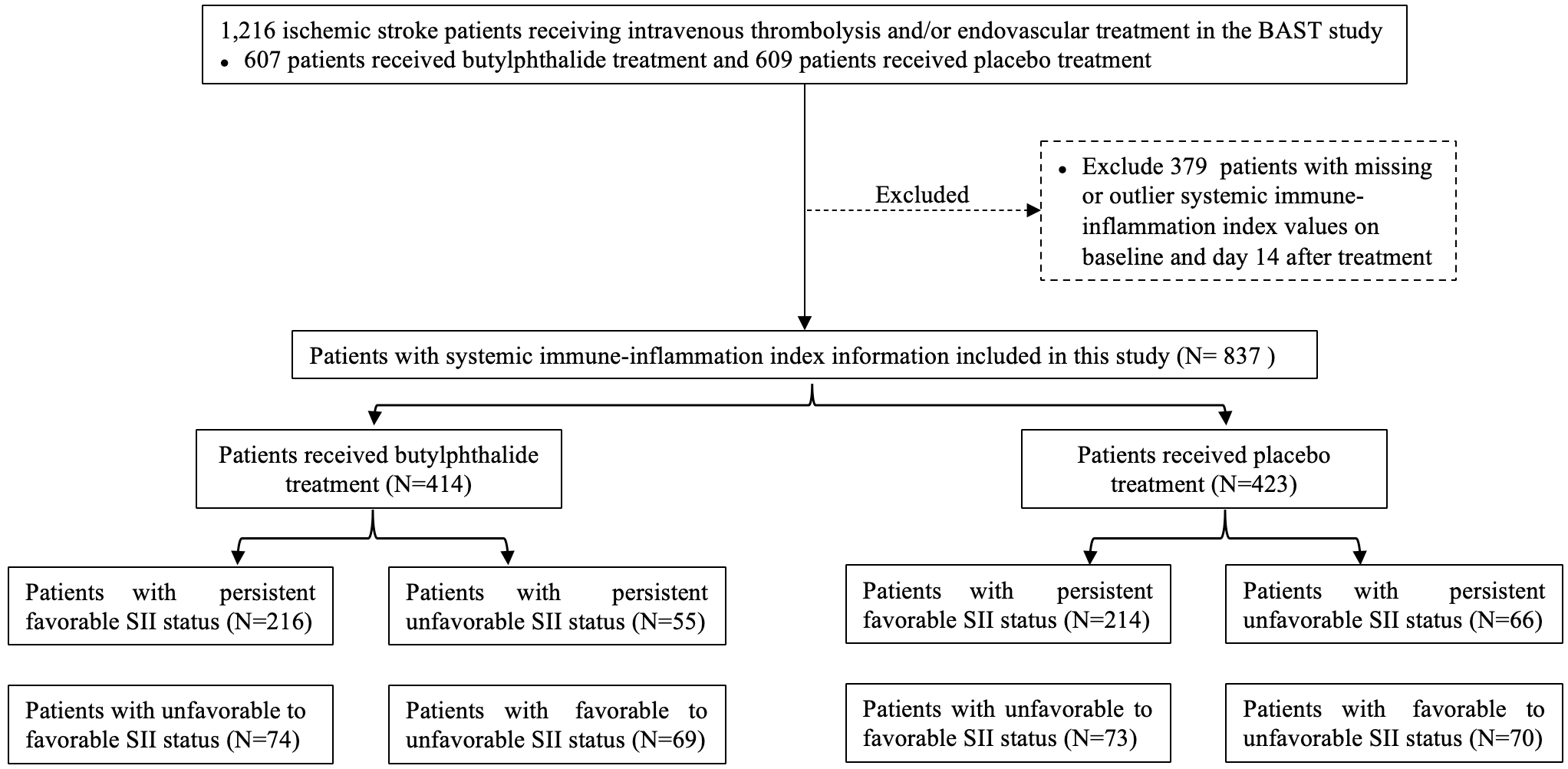


**Figure S1**. Selection process of the study population

Abbreviation: BAST: the Butylphthalide for Acute Ischemic Stroke Patients Receiving Intravenous Thrombolysis or Endovascular Treatment.

A

B

D

C


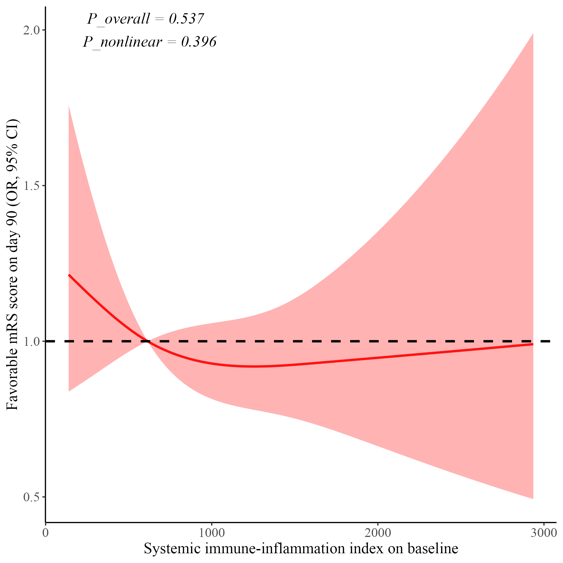

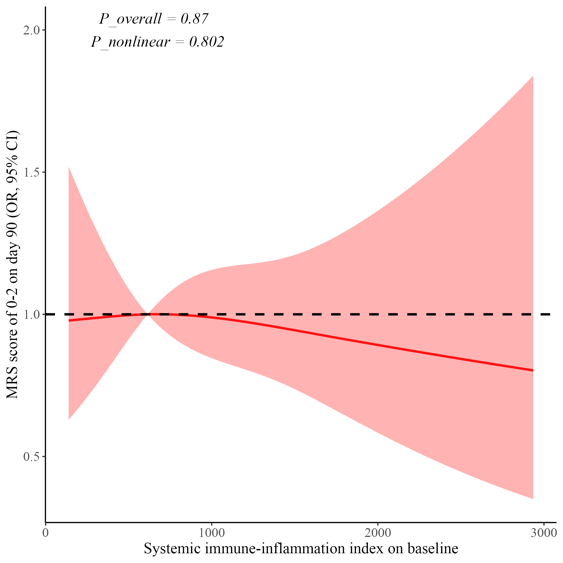

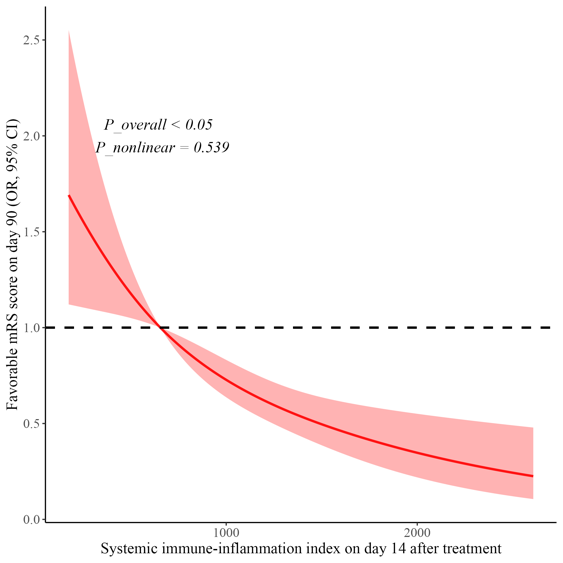

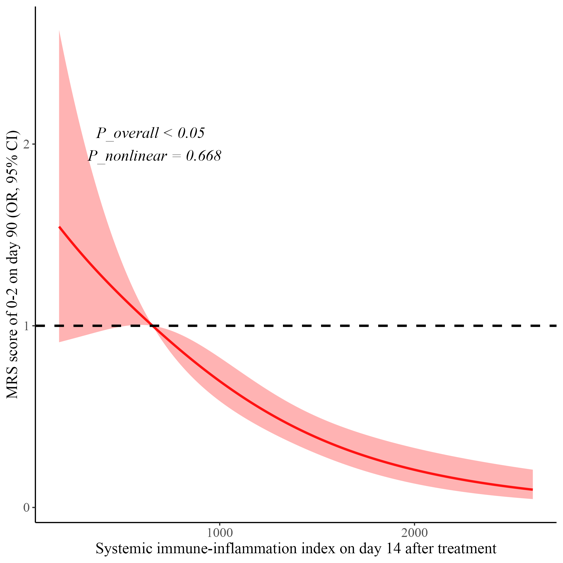


**Figure S2.** Restricted cubic splines of the systemic immune-inflammation index for favorable mRS and mRS score of 0-2 on day 90

A: Restricted cubic splines of the systemic immune-inflammation index at baseline for favorable mRS score on day 90; B: Restricted cubic splines of the systemic immune-inflammation index at 14 days after treatment for favorable mRS score on day 90; C: Restricted cubic splines of the systemic immune-inflammation index at baseline for mRS score of 0-2 on day 90; D: Restricted cubic splines of the systemic immune-inflammation index at 14 days after treatment for mRS score of 0-2 on day 90.


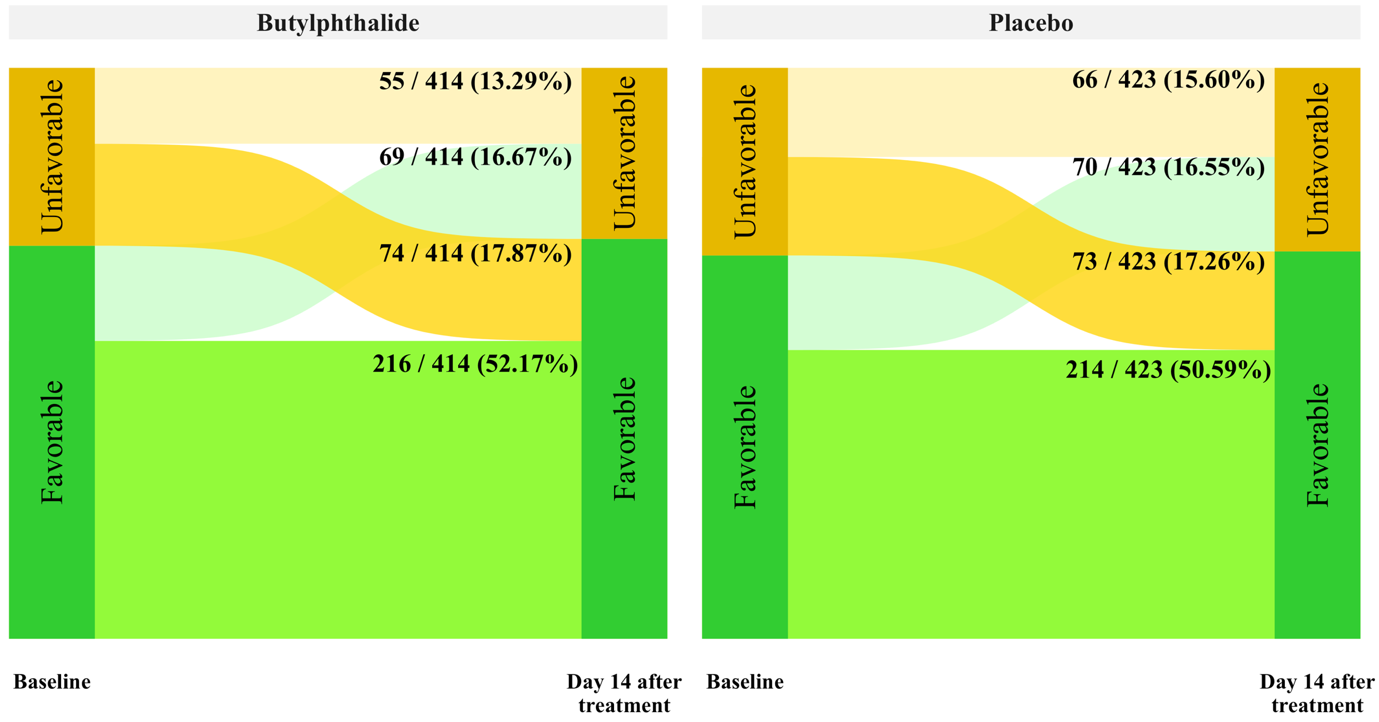


**Figure S****3.** Four SII change patterns during acute phase of ischemic stroke stratified by treatment

**Table S1**. Baseline characteristics of included and excluded patients

| Characteristics | Included patients (N=837) | Excluded patients (N=379) | *P* value |
| --- | --- | --- | --- |
| Age, median (IQR), y | 66.0 (56.0, 72.0) | 66.0 (57.0, 72.0) | 0.590 |
| BMI, kg/m^2^, median (IQR) | 24.2 (22.0, 26.4) | 24.2 (22.0, 26.7) | 0.990 |
| SBP, mmHg, median (IQR) | 150.0 (137.0, 163.5) | 150.0 (137.5, 162.5) | 0.880 |
| NIHSS score, median (IQR) | 8.0 (5.0, 12.0) | 10.0 (6.0, 14.0) | < 0.001 |
| Female | 265 (31.7%) | 124 (32.7%) | 0.764 |
| mRS score prior to onset=0 | 780 (93.2%) | 356 (93.9%) | 0.720 |
| Stroke history | 191 (22.8%) | 90 (23.7%) | 0.778 |
| Heart disease history | 197 (23.5%) | 94 (24.8%) | 0.684 |
| Hypertension history | 495 (59.1%) | 201 (53.0%) | 0.054 |
| Revascularization treatment |  |  | 0.210 |
| Endovascular or bridging | 251 (30.0%) | 128 (33.8%) |  |
| Intravenous rt-PA treatment | 586 (70.0%) | 251 (66.2%) |  |
| TOAST subtype |  |  | 0.498 |
| LAA | 419 (50.1%) | 195 (51.5%) |  |
| CE | 156 (18.6%) | 66 (17.4%) |  |
| SAA | 233 (27.8%) | 98 (25.9%) |  |
| SOE | 13 (1.6%) | 11 (2.9%) |  |
| SUE | 16 (1.9%) | 9 (2.4%) |  |

IQR: interquartile range; SBP: systolic blood pressure; NIHSS: national institutes of health stroke scale; LAA: large-artery atherosclerosis; CE: cardioembolism; SAA: small-artery occlusion lacunar; SOE: stroke of other determined etiology; SUE: stroke of undetermined etiology.

**Table S2**. Proportional hazards assumption test results for the Cox model of systemic immune inflammation related indicator with outcomes

| Model term | Recurrent symptomatic stroke within 90 days | Combined vascular events within 90 days | Death within 90 days | Recurrent symptomatic ischemic stroke within 90 days |
| --- | --- | --- | --- | --- |
| Systemic immune inflammation change pattern | | |  |  |
| Chi-square statistic | 9.76 | 7.65 | 31.52 | 17.58 |
| P value | 0.637 | 0.812 | 0.002 | 0.129 |
|  |  |  |  |  |
| Systemic immune inflammation on Day 14 | | |  |  |
| Chi-square statistic | 8.74 | 7.39 | 27.36 | 17.62 |
| P value | 0.557 | 0.688 | 0.002 | 0.062 |
|  |  |  |  |  |
| SII change pattern–medication combination | | | | |
| Chi-square statistic | 11.05 | 7.97 | - | - |
| P value | 0.749 | 0.925 | - | - |

| Functional outcomes | Systemic immune inflammation change pattern | Events / Total  (Proportion) | Adjusted effect | | |
| --- | --- | --- | --- | --- | --- |
|  |  |  | Effect (95% CI) | P value | P _FDR_ value |
| Favorable mRS score on day 90 | Unfavorable on day 14 | 94 / 260 (36.2%) | OR, 1 [Reference] |  |  |
|  | Favorable on day 14 | 349 / 577 (60.5%) | **2.8 (2.01, 3.90)** | **< 0.001** | **< 0.001** |
| mRS score of 0-2 on day 90 | Unfavorable on day 14 | 147 / 260 (56.5%) | OR, 1 [Reference] |  |  |
|  | Favorable on day 14 | 491 / 577 (85.1%) | **3.62 (2.51, 5.21)** | **< 0.001** | **< 0.001** |
| Recurrent symptomatic stroke within 90 days | Unfavorable on day 14 | 38 / 260 (14.6%) | HR, 1 [Reference] |  |  |
|  | Favorable on day 14 | 30 / 577 (5.2%) | **0.31 (0.18, 0.51)** | **< 0.001** | **< 0.001** |
| Combined vascular events within 90 days | Unfavorable on day 14 | 42 / 260 (16.2%) | HR, 1 [Reference] |  |  |
|  | Favorable on day 14 | 33 / 577 (5.7%) | **0.33 (0.20, 0.53)** | **< 0.001** | **< 0.001** |
| Death within 90 days | Unfavorable on day 14 | 19 / 260 (7.3%) | HR, 1 [Reference] |  |  |
|  | Favorable on day 14 | 9 / 577 (1.6%) | **0.3 (0.14, 0.61)** | **0.001** | **0.001** |
| Recurrent symptomatic ischemic stroke within 90 days | Unfavorable on day 14 | 18 / 260 (6.9%) | HR, 1 [Reference] |  |  |
|  | Favorable on day 14 | 22 / 577 (3.8%) | **0.49 (0.26, 0.94)** | **0.032** | **0.032** |

**Table S3**. Associations of day-14 systemic immune-inflammation status with 90-day functional outcomes in patients with ischemic stroke

The analysis adjusted baseline covariates including treatment (butylphthalide vs placebo), age, sex, body mass index, pre-onset modified Rankin Scale score, National Institutes of Health Stroke Scale score, and relevant medical histories (stroke, heart disease, hypertension).

OR: odds ratio; HR: hazard ratio; P _FDR_ value: false discovery rate-adjusted P-value; IQR: interquartile range.

Boldface type indicates effect estimates were statistically signiﬁcant, P_FDR_ value < 0.05.

**Table S4**. Associations of butylphthalide treatment with 90-day functional outcomes across different systemic immune-inflammation change patterns

| Functional outcomes | Systemic immune inflammation changes patterns | Placebo | Butylphthalide | Adjusted effect | | | P for interaction |
| --- | --- | --- | --- | --- | --- | --- | --- |
|  |  | Events / Total  (Proportion) | Events / Total  (Proportion) | OR (95% CI) | P value | P _FDR_ value |  |
| Favorable mRS score on day 90 | Persistent unfavorable | 24 / 66 (36.4%) | 21 / 55 (38.2%) | 1.07 (0.46, 2.48) | 0.878 | 0.878 | 0.625 |
|  | Favorable−unfavorable | 18 / 70 (25.7%) | 31 / 69 (44.9%) | 2.22 (1.01, 4.89) | 0.047 | 0.145 |  |
|  | Unfavorable−favorable | 42 / 73 (57.5%) | 52 / 74 (70.3%) | 2.01 (0.91, 4.44) | 0.085 | 0.156 |  |
|  | Persistent favorable | 112 / 214 (52.3%) | 143 / 216 (66.2%) | **1.83 (1.21, 2.77)** | **0.004** | **0.044** |  |
| mRS score of 0-2 on day 90 | Persistent unfavorable | 37 / 66 (56.1%) | 35 / 55 (63.6%) | 1.34 (0.55, 3.26) | 0.524 | 0.576 | 0.734 |
|  | Favorable−unfavorable | 32 / 70 (45.7%) | 43 / 69 (62.3%) | 2.22 (0.99, 4.96) | 0.053 | 0.145 |  |
|  | Unfavorable−favorable | 59 / 73 (80.8%) | 64 / 74 (86.5%) | 1.55 (0.54, 4.48) | 0.418 | 0.511 |  |
|  | Persistent favorable | 178 / 214 (83.2%) | 190 / 216 (88.0%) | 1.72 (0.97, 3.07) | 0.066 | 0.145 |  |
| Recurrent symptomatic stroke within 90 days | Persistent unfavorable | 12 / 66 (18.2%) | 7 / 55 (12.7%) | 0.70 (0.23, 2.07) | 0.515 | 0.800 | 0.007 |
|  | Favorable−unfavorable | 8 / 70 (11.4%) | 11 / 69 (15.9%) | 1.37 (0.49, 3.84) | 0.55 | 0.800 |  |
|  | Unfavorable−favorable | 3 / 73 (4.1%) | 7 / 74 (9.5%) | 3.39 (0.83, 13.82) | 0.089 | 0.428 |  |
|  | Persistent favorable | 15 / 214 (7.0%) | 5 / 216 (2.3%) | 0.32 (0.11, 0.89) | 0.029 | 0.428 |  |
| Combined vascular events within 90 days | Persistent unfavorable | 14 / 66 (21.2%) | 7 / 55 (12.7%) | 0.60 (0.21, 1.68) | 0.329 | 0.791 | 0.010 |
|  | Favorable−unfavorable | 10 / 70 (14.3%) | 11 / 69 (15.9%) | 1.07 (0.41, 2.79) | 0.892 | 0.947 |  |
|  | Unfavorable−favorable | 3 / 73 (4.1%) | 8 / 74 (10.8%) | 3.66 (0.93, 14.39) | 0.064 | 0.428 |  |
|  | Persistent favorable | 15 / 214 (7.0%) | 7 / 216 (3.2%) | 0.43 (0.17, 1.08) | 0.073 | 0.428 |  |

The analysis adjusted baseline covariates including age, sex, body mass index, pre-onset modified Rankin Scale score, National Institutes of Health Stroke Scale score, and relevant medical histories (stroke, heart disease, hypertension).

OR: odds ratio; HR: hazard ratio; P _FDR_ value: false discovery rate-adjusted P-value; IQR: interquartile range.

Boldface type indicates effect estimates were statistically signiﬁcant, P_FDR_ value < 0.05.

**Table S5**. Associations of systemic immune inflammation change patterns with a favorable mRS score on day 90 stratified by reperfusion modality

| Revascularization treatment | Systemic immune inflammation changes patterns | Events / Total  (Proportion) | Adjusted effect | | | P for Interaction |
| --- | --- | --- | --- | --- | --- | --- |
|  |  |  | OR (95% CI) | P value | P _FDR_ value |  |
| Endovascular treatment or bridging | Persistent unfavorable | 25 / 53 (47.2%) | OR, 1 [Reference] |  |  | 0.321 |
|  | Favorable−unfavorable | 25 / 60 (41.7%) | 0.76 (0.34, 1.70) | 0.502 | 0.602 |  |
|  | Unfavorable−favorable | 41 / 50 (82.0%) | **5.66 (2.07, 15.43)** | **< 0.001** | **< 0.001** |  |
|  | Persistent favorable | 60 / 88 (68.2%) | 2.18 (1.02, 4.65) | 0.044 | 0.066 |  |
| Intravenous rt-PA treatment | Persistent unfavorable | 20 / 68 (29.4%) | OR, 1 [Reference] |  |  |  |
|  | Favorable−unfavorable | 24 / 79 (30.4%) | 1.03 (0.49, 2.15) | 0.938 | 0.938 |  |
|  | Unfavorable−favorable | 53 / 97 (54.6%) | **2.55 (1.28, 5.08)** | **0.008** | **0.016** |  |
|  | Persistent favorable | 195 / 342 (57.0%) | **2.97 (1.65, 5.38)** | **< 0.001** | **< 0.001** |  |

The analysis adjusted baseline covariates including age, sex, body mass index, pre-onset modified Rankin Scale score, National Institutes of Health Stroke Scale score, and relevant medical histories (stroke, heart disease, hypertension).

OR: odds ratio; P _FDR_ value: false discovery rate-adjusted P-value

Boldface type indicates effect estimates were statistically signiﬁcant, P_FDR_ value < 0.05.

**Table S6**. Associations of butylphthalide treatment with 90-day functional outcomes by day-14 systemic immune-inflammation status

| Functional outcomes | Systemic immune-inflammation status | Placebo | Butylphthalide | Adjusted effect | | | P for Interaction |
| --- | --- | --- | --- | --- | --- | --- | --- |
|  |  | Events / Total  (Proportion) | Events / Total  (Proportion) | Effect (95% CI) | P value | P _FDR_ value |  |
| Favorable mRS score on day 90 | Unfavorable on day 14 | 42 / 136 (30.9%) | 52 / 124 (41.9%) | 1.46 (0.85, 2.52) | 0.171 | 0.228 | 0.556 |
|  | Favorable on day 14 | 154 / 287 (53.7%) | 195 / 290 (67.2%) | **1.83 (1.28, 2.63)** | **< 0.001** | **0.012** |  |
| mRS score of 0-2 on day 90 | Unfavorable on day 14 | 69 / 136 (50.7%) | 78 / 124 (62.9%) | 1.52 (0.86, 2.66) | 0.148 | 0.222 | 0.958 |
|  | Favorable on day 14 | 237 / 287 (82.6%) | 254 / 290 (87.6%) | 1.58 (0.97, 2.57) | 0.064 | 0.147 |  |
| Recurrent symptomatic stroke within 90 days | Unfavorable on day 14 | 20 / 136 (14.7%) | 18 / 124 (14.5%) | 0.84 (0.43, 1.66) | 0.618 | 0.800 | 0.525 |
|  | Favorable on day 14 | 18 / 287 (6.3%) | 12 / 290 (4.1%) | 0.68 (0.33, 1.42) | 0.304 | 0.791 |  |
| Combined vascular events within 90 days | Unfavorable on day 14 | 24 / 136 (17.6%) | 18 / 124 (14.5%) | 0.70 (0.37, 1.35) | 0.289 | 0.791 | 0.879 |
|  | Favorable on day 14 | 18 / 287 (6.3%) | 15 / 290 (5.2%) | 0.83 (0.42, 1.65) | 0.594 | 0.800 |  |
| Death within 90 days | Unfavorable on day 14 | 14 / 136 (10.3%) | 5 / 124 (4.0%) | 0.60 (0.20, 1.82) | 0.369 | 0.800 | 0.677 |
|  | Favorable on day 14 | 5 / 287 (1.7%) | 4 / 290 (1.4%) | 0.74 (0.19, 2.88) | 0.661 | 0.800 |  |
| Recurrent symptomatic ischemic stroke within 90 days | Unfavorable on day 14 | 7 / 136 (5.1%) | 11 / 124 (8.9%) | 1.44 (0.53, 3.92) | 0.472 | 0.800 | 0.605 |
|  | Favorable on day 14 | 10 / 287 (3.5%) | 12 / 290 (4.1%) | 1.28 (0.55, 2.98) | 0.563 | 0.800 |  |

The analysis adjusted baseline covariates including age, sex, body mass index, pre-onset modified Rankin Scale score, National Institutes of Health Stroke Scale score, and relevant medical histories (stroke, heart disease, hypertension).

OR: odds ratio; HR: hazard ratio; P _FDR_ value: false discovery rate-adjusted P-value; IQR: interquartile range.

Boldface type indicates effect estimates were statistically signiﬁcant, P_FDR_ value < 0.05.


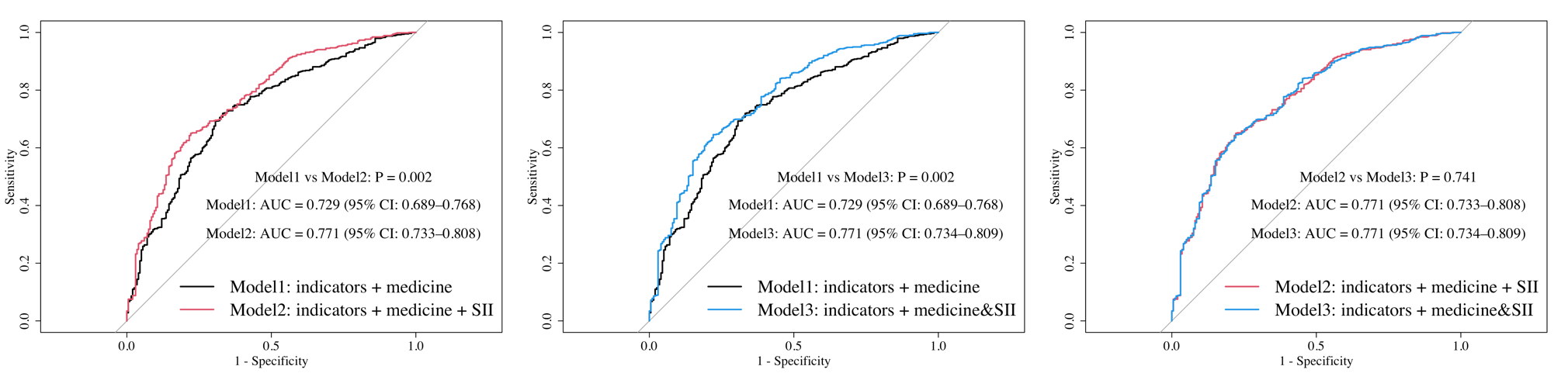

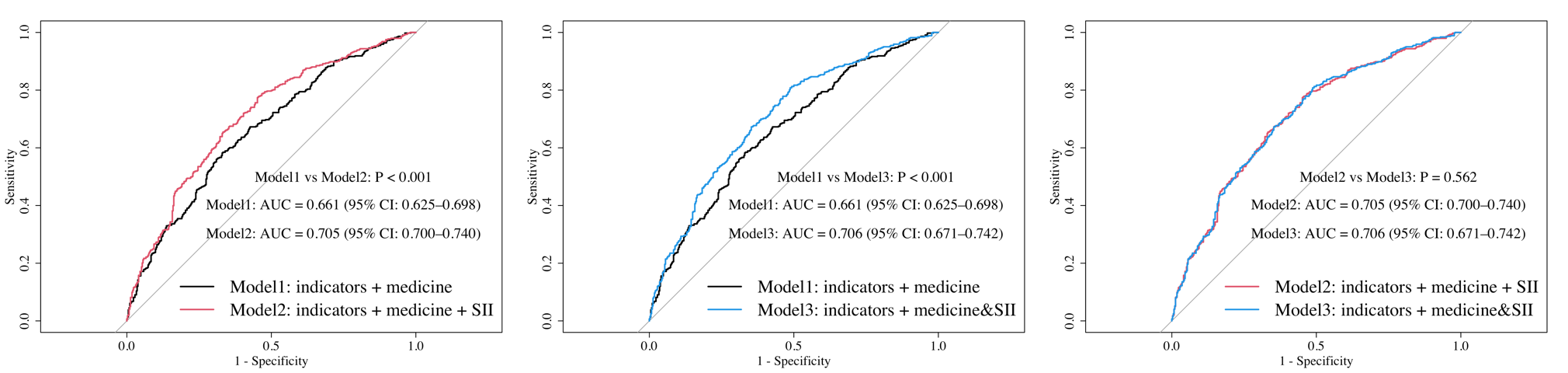


B

A

**Figure S4**. ROC curve analysis of regression models for evaluating the predictive performance enhancement by the systemic immune-inflammation index change patterns

A: ROC curve analysis of regression models for predicting favorable mRS score on day 90; B: ROC curve analysis of regression models for predicting mRS score of 0-2 on day 90. Indicators included age, sex, body mass index, pre-onset mRS, baseline NIHSS, and relevant medical histories (stroke, heart disease, hypertension). Medicine referred to butylphthalide treatment or placebo treatment. medicine&SII referred to combination of butylphthalide and SII change pattern. SII: systemic immune-inflammation index.


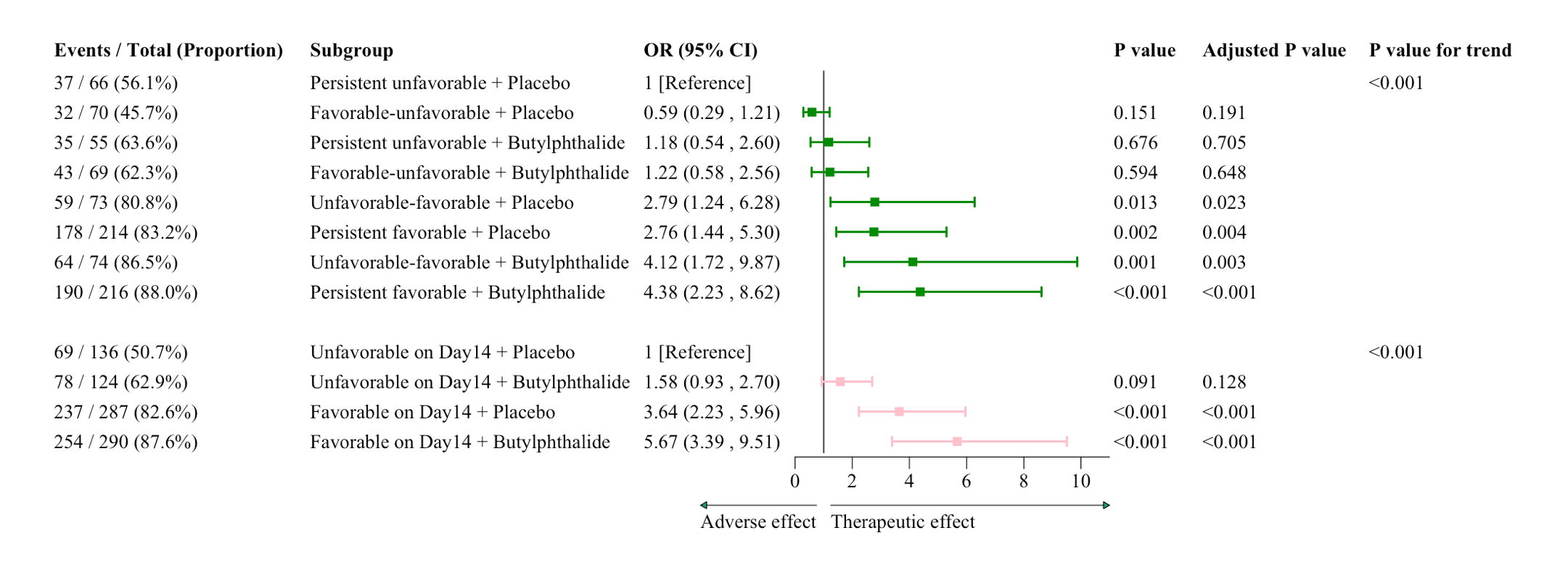


**Figure S5.** Joint associations of butylphthalide and systemic immune-inflammation index change pattern with a mRS score of 0-2 on day 90

In the analysis examining the exploratory joint associations of butylphthalide treatment and SII change patterns with mRS score of 0-2 on day 90, the "Persistent unfavorable + Placebo" pattern, defined as patients who received placebo and had a persistent unfavorable SII pattern during the acute phase of ischemic stroke, served as the reference category. In the analysis examining joint associations of butylphthalide and SII status on day 14 post-treatment with mRS score of 0-2 on day 90, the "Unfavorable + Placebo" pattern, defined as patients who received placebo and had unfavorable SII status on day 14 after treatment, served as the reference category.

Both analyses were adjusted for baseline covariates, including age, sex, body mass index, pre-onset modified Rankin Scale score, National Institutes of Health Stroke Scale score, and relevant medical histories (stroke, heart disease, hypertension).

OR: odds ratio; P _FDR_ value: false discovery rate-adjusted P-value.


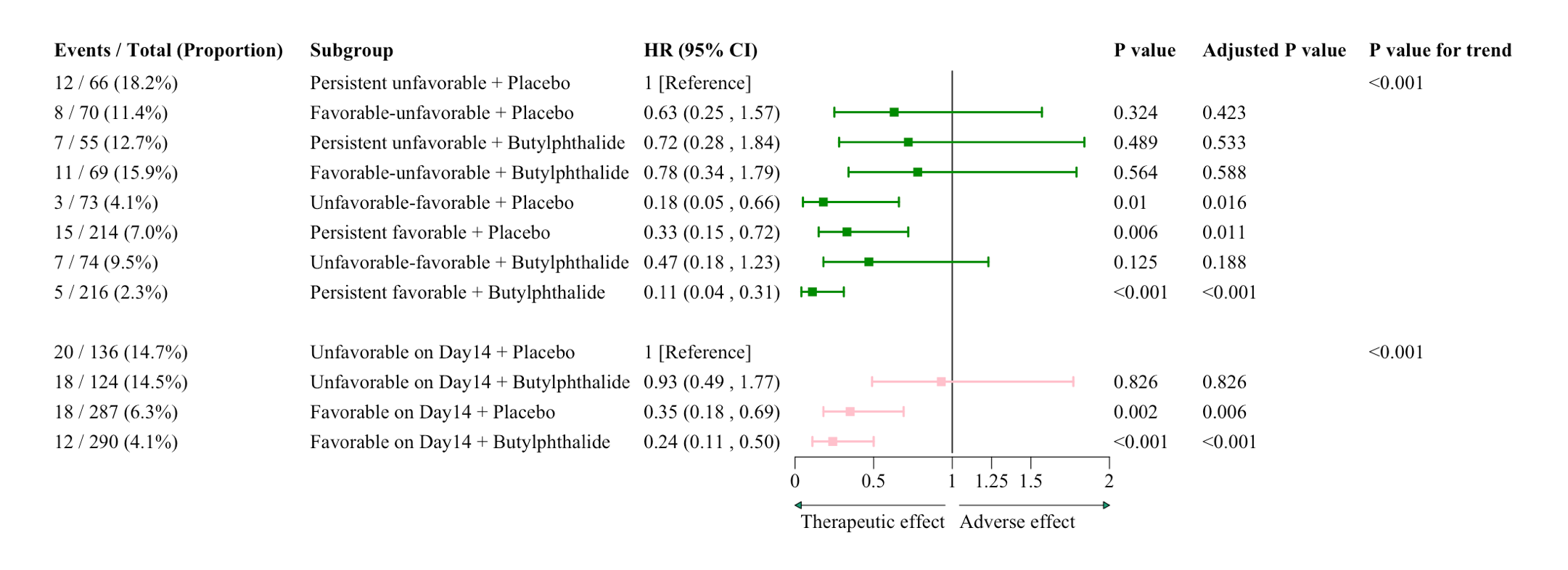


**Figure** **S6.** Joint associations of butylphthalide and systemic immune-inflammation index change pattern with recurrent symptomatic stroke within 90 days

In the analysis examining the exploratory joint associations of butylphthalide treatment and SII change patterns with recurrent symptomatic stroke within 90 days, the “Persistent unfavorable + Placebo” group, defined as patients who received placebo and had a persistent unfavorable SII pattern during the acute phase of ischemic stroke, served as the reference category. In the analysis examining joint associations of butylphthalide and SII status on day 14 post-treatment with recurrent symptomatic stroke within 90 days, the “Unfavorable + Placebo” group, defined as patients who received placebo and had unfavorable SII status on day 14 after treatment, served as the reference category.

Both analyses were adjusted for baseline covariates, including age, sex, body mass index, pre-onset modified Rankin Scale score, National Institutes of Health Stroke Scale score, and relevant medical histories (stroke, heart disease, hypertension).

HR: hazard ratio; P _FDR_ value: false discovery rate-adjusted P-value.


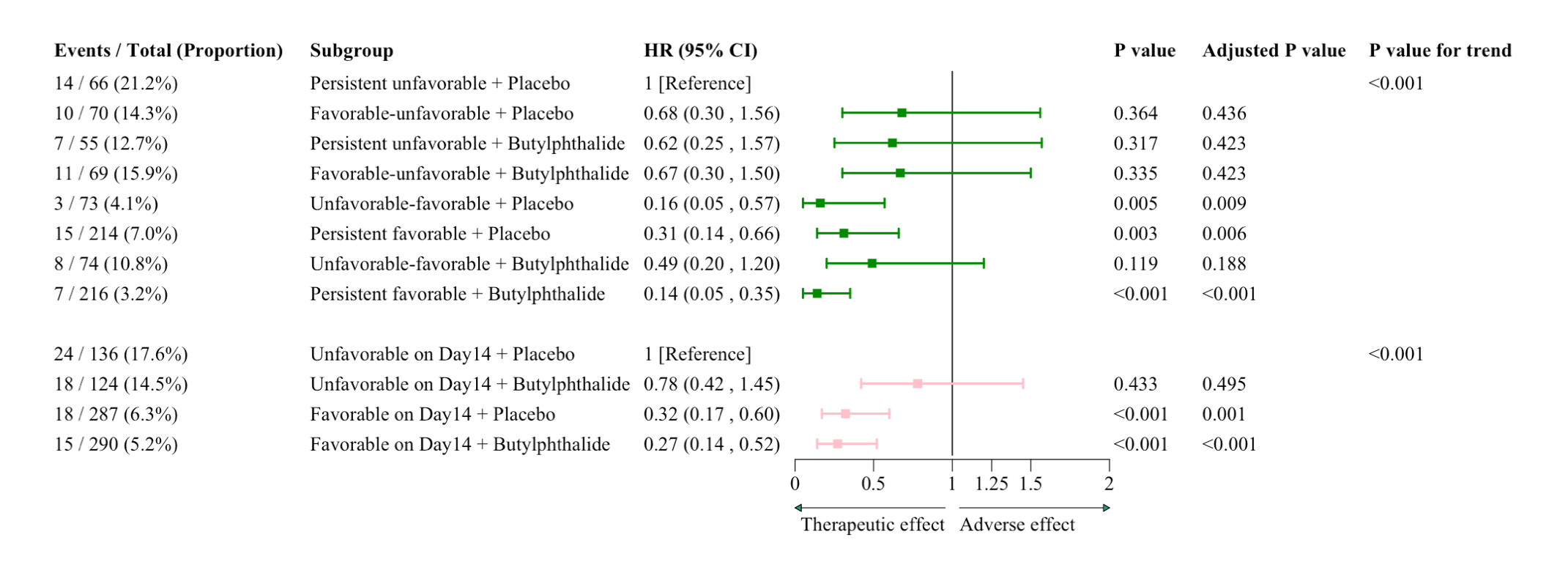


**F****igure S7**. Joint associations of butylphthalide and systemic immune-inflammation index change pattern with combined vascular events within 90 days

In the analysis examining the exploratory joint associations of butylphthalide treatment and SII change patterns with combined vascular events within 90 days, the "Persistent unfavorable + Placebo" pattern, defined as patients who received placebo and had a persistent unfavorable SII pattern during the acute phase of ischemic stroke, served as the reference category. In the analysis examining joint associations of butylphthalide and SII status on day 14 post-treatment with combined vascular events within 90 days, the "Unfavorable + Placebo" pattern, defined as patients who received placebo and had unfavorable SII status on day 14 after treatment, served as the reference category.

Both analyses were adjusted for baseline covariates, including age, sex, body mass index, pre-onset modified Rankin Scale score, National Institutes of Health Stroke Scale score, and relevant medical histories (stroke, heart disease, hypertension).

HR: hazard ratio; P _FDR_ value: false discovery rate-adjusted P-value.

***
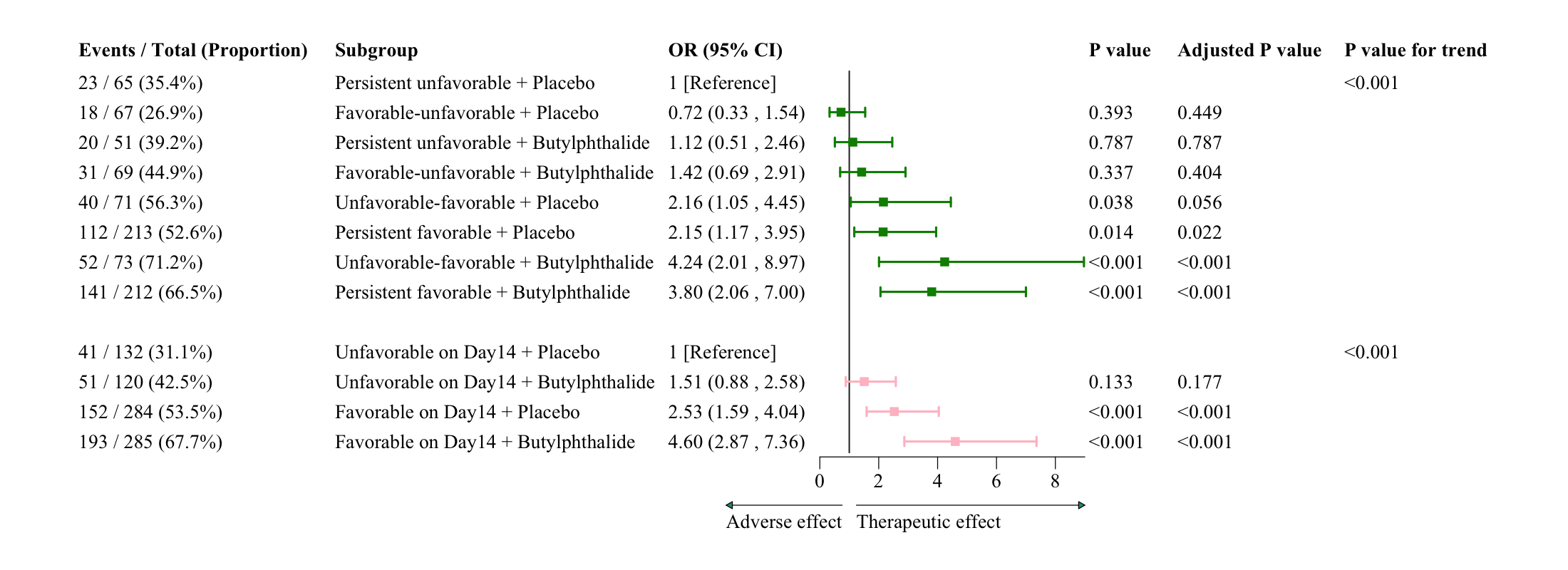
***

**Figure S8**. Sensitivity analysis of the joint associations of butylphthalide and systemic immune-inflammation index change patterns with a favorable 90-day mRS scores in ischemic stroke patients additionally excluding individuals with acute infection or immune system diseases

In the analysis examining the exploratory joint associations of butylphthalide treatment and SII change patterns with favorable mRS score on day 90, the "Persistent unfavorable + Placebo" pattern, defined as patients who received placebo and had a persistent unfavorable SII pattern during the acute phase of ischemic stroke, served as the reference category. In the analysis examining joint associations of butylphthalide and SII status on day 14 post-treatment with favorable mRS score on day 90, the "Unfavorable + Placebo" pattern, defined as patients who received placebo and had unfavorable SII status on day 14 after treatment, served as the reference category.

Both analyses were adjusted for baseline covariates, including age, sex, body mass index, pre-onset modified Rankin Scale score, National Institutes of Health Stroke Scale score, and relevant medical histories (stroke, heart disease, hypertension).

OR: odds ratio; P _FDR_ value: false discovery rate-adjusted P-value.


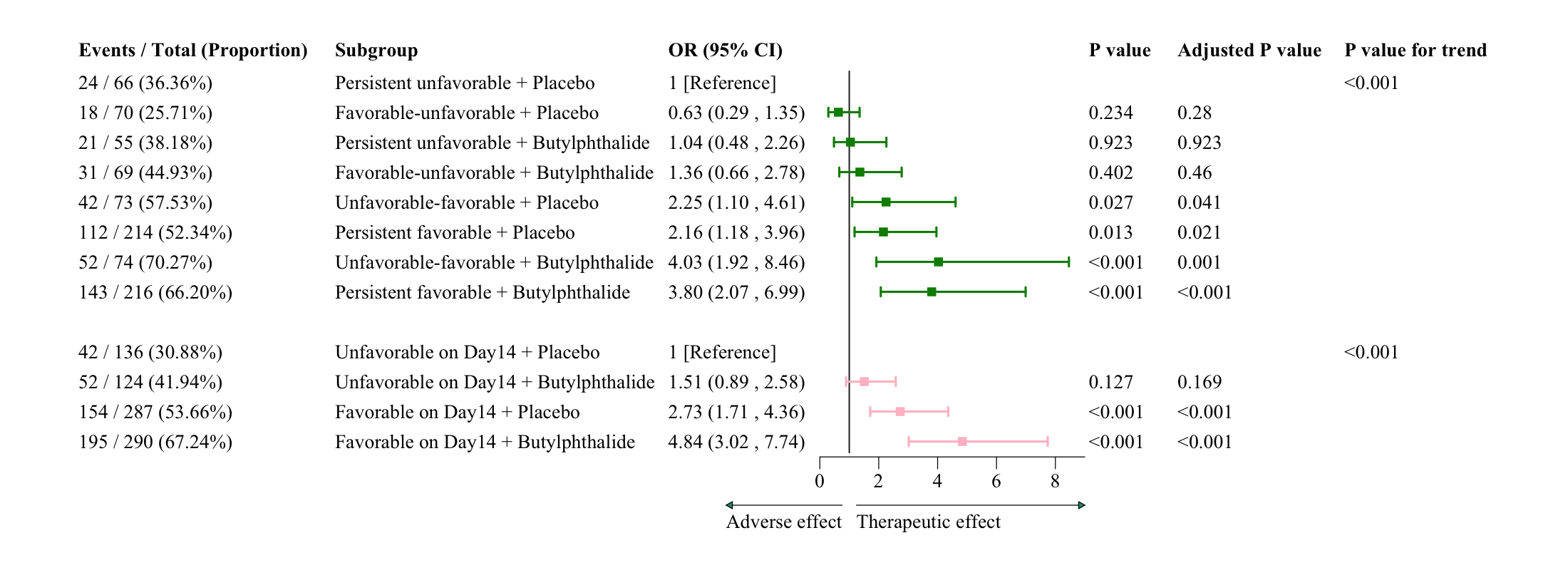


**Figure S9**. Sensitivity analysis of the joint associations of butylphthalide and SII change patterns with a favorable 90-day mRS scores in ischemic stroke patients additionally adjusting reperfusion modality

In the analysis examining the exploratory joint associations of butylphthalide treatment and SII change patterns with favorable mRS score on day 90, the "Persistent unfavorable + Placebo" pattern, defined as patients who received placebo and had a persistent unfavorable SII pattern during the acute phase of ischemic stroke, served as the reference category. In the analysis examining joint associations of butylphthalide and SII status on day 14 post-treatment with favorable mRS score on day 90, the "Unfavorable + Placebo" pattern, defined as patients who received placebo and had unfavorable SII status on day 14 after treatment, served as the reference category.

Both analyses were adjusted for baseline covariates, including age, sex, body mass index, pre-onset modified Rankin Scale score, National Institutes of Health Stroke Scale score, reperfusion modality and relevant medical histories (stroke, heart disease, hypertension).

OR: odds ratio; P _FDR_ value: false discovery rate-adjusted P-value.


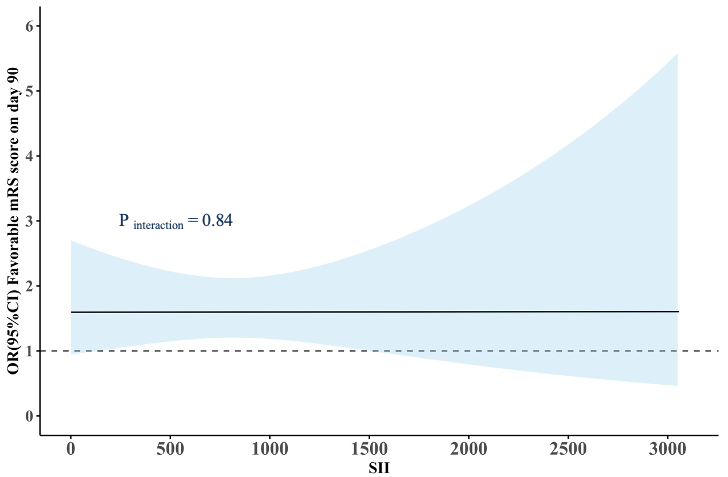


B

A


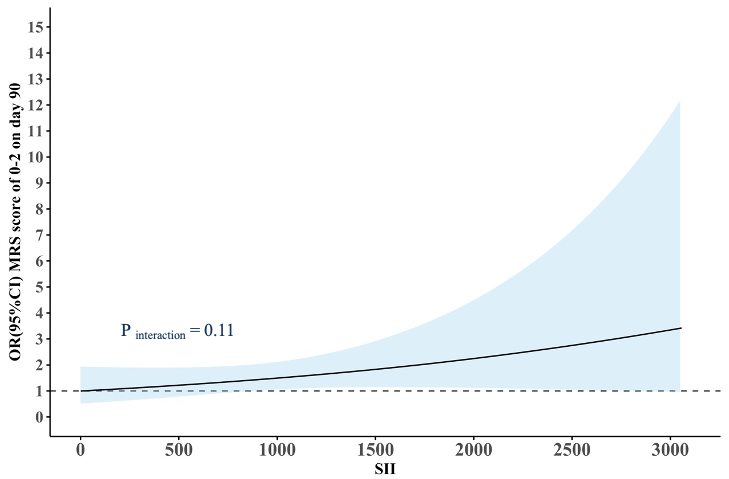


**Figure S10**. Interaction between baseline SII value and butylphthalide treatment on 90-day functional outcomes

A. Interaction between baseline SII value and butylphthalide treatment on favorable mRS score on day 90; B. Interaction between baseline SII value and butylphthalide treatment on mRS score of 0-2 on day 90.

**Table S7**. Detailed information on identification of gene targets

| Butylphthalide | Datasets | Coremine | ChEMBL | TargetNet | SwissTargetPrediction | PharmMapper |
| --- | --- | --- | --- | --- | --- | --- |
|  | Search Query | Butylphthalide OR 3-n-butylphthalide | SMILES | SMILES | SMILES | SMILES |
|  | Screening criteria | Deduplication | None | Probability >0.001 | Probability> 0.03 | Norm fit>0.9  Homo sapiens |
|  | Number of genes | 207 | 132 | 147 | 42 | 10 |
| Ischemic stroke | Datasets | CTD | GeneCards | TTD | OMIM |  |
|  | Search Query | ischemic stroke | ischemic stroke | ischemic stroke | ischemic stroke |  |
|  | Screening criteria | Inference Score>20 | Relevance score >10 | None | None |  |
|  | Number of genes | 1323 | 571 | 29 | 14 |  |
| Inflammation | Datasets | CTD | GeneCards | TTD | OMIM |  |
|  | Search Query | inflammation | inflammation | inflammation | inflammation |  |
|  | Screening criteria | Inference Score>100 | Relevance score >10 | None | None |  |
|  | Number of genes | 10116 | 219 | 163 | 13 |  |

Abbreviations: CTD: comparative Toxicogenomics Database; TTD: Therapeutic Target Database; SMILES of butylphthalide: CCCCC1C2=CC=CC=C2C(=O)O1; List of database websites: Coremine database: <https://www.coremine.com/medical/#search> ; ChEMBL: <https://www.ebi.ac.uk/chembl/> ; TargetNet: <http://targetnet.scbdd.com/>; SwissTargetPrediction: <https://swisstargetprediction.ch/> ; PharmMapper: <http://59.78.96.61/pharmmapper> ; Comparative Toxicogenomics Database: <https://ctdbase.org/>; GeneCards: <https://www.genecards.org/>; Therapeutic Target Database: <https://ttd.idrblab.cn/>; OMIM: <https://omim.org/>

Reference

1. Ma F, Li L, Xu L, Wu J, Zhang A, Liao J, et al. The relationship between systemic inflammation index, systemic immune-inflammatory index, and inflammatory prognostic index and 90-day outcomes in acute ischemic stroke patients treated with intravenous thrombolysis. J Neuroinflammation 2023; 20 (1): 220. doi: 10.1186/s12974-023-02890-y.

2. Jiang Y, Cui Y, Hu X, Lian J, Qin X, Wang X, et al. Prognostic assessment of acute ischemic stroke by systemic immune-inflammatory index: a comprehensive meta-analysis of multidimensional outcomes. Front Neurol 2025; 16 1594258. doi: 10.3389/fneur.2025.1594258.

3. Fisher LD, Lin DY. Time-dependent covariates in the Cox proportional-hazards regression model. Annu Rev Public Health 1999; 20 145-157. doi: 10.1146/annurev.publhealth.20.1.145.

4. Kim Y, Jeong HS, Shim SR, Hyun JK. Discharge Neurological Deficit as a Predictor of Early Stroke Recurrence: A Nationwide Registry‐Based Propensity‐Matched Study. Journal of the American Heart Association 2025; 14 (20): e043529. doi:10.1161/JAHA.125.043529.

5. de Havenon A, Viscoli C, Kleindorfer D, Sucharew H, Delic A, Becker C, et al. Disability and Recurrent Stroke Among Participants in Stroke Prevention Trials. JAMA Netw Open 2024; 7 (7): e2423677. doi: 10.1001/jamanetworkopen.2024.23677.

6. Liu X, Zhang D, Liu Y, Sun X, Hou Y, Wang B, et al. A J-shaped relation of BMI and stroke: Systematic review and dose-response meta-analysis of 4.43 million participants. Nutr Metab Cardiovasc Dis 2018; 28 (11): 1092-1099. doi: 10.1016/j.numecd.2018.07.004.

7. Liu H, Wang P, Wu LH, Wu F, Zhou X, Li Y, et al. Evaluating Immune-Inflammatory Indices for Risk Stratification in Cardiovascular Disease: An Umbrella Review of Systematic Reviews and Meta-Analyses. Diagnostics (Basel) 2025; 15 (22): doi: 10.3390/diagnostics15222862.

8. Yang CS, Guo A, Li Y, Shi K, Shi FD, Li M. Dl-3-n-butylphthalide Reduces Neurovascular Inflammation and Ischemic Brain Injury in Mice. Aging Dis 2019; 10 (5): 964-976. doi: 10.14336/ad.2019.0608.
